# Pectolinarigenin Attenuates LPS-Induced Lung Inflammation and Injury with Reduced HDAC3/NF-κB/NLRP3 Signaling

**DOI:** 10.3390/antiox15070898

**Published:** 2026-07-20

**Authors:** Danbee Kim, Dong-Keon Lee, Jeong-Ran Park

**Affiliations:** 1 Division of Research Program, Scripps Korea Antibody Institute, Chuncheon 24341, Gangwon-do, Republic of Korea; kdb0515@skai.or.kr (D.K.); jocker81@wku.ac.kr (D.-K.L.); 2 Department of Biochemistry, School of Medicine, Wonkwang University, Iksan-Daero 460, Iksan-Si 54538, Jeonbuk-Do, Republic of Korea

**Keywords:** pectolinarigenin, acute lung injury, inflammasome, NLRP3, HDAC3, NF-κB

## Abstract

Pectolinarigenin (PEC), a naturally occurring flavonoid, exhibits anti-inflammatory and antioxidant activities in various experimental models. However, its protective effects against lipopolysaccharide (LPS)-induced lung inflammation and the underlying molecular mechanisms remain unclear. This study investigated the protective effects of PEC using LPS-treated MLE12 cells and RAW264.7 macrophages, as well as a prophylactic mouse model in which PEC was administered before LPS exposure. In LPS-treated MLE12 cells and RAW264.7 macrophages, PEC reduced inflammatory responses and cellular injury, accompanied by decreased reactive oxygen species production and modulation of the histone deacetylase 3 (HDAC3)/nuclear factor κB (NF-κB)/NOD-like receptor family pyrin domain-containing protein 3 (NLRP3) signaling. Consistent with these findings, PEC pretreatment attenuated pulmonary edema, inflammatory cell infiltration, pro-inflammatory cytokine production, oxidative stress, pyroptosis-associated signaling, and histopathological lung injury in LPS-exposed mice. These protective effects were accompanied by reduced HDAC3 expression and nuclear localization, together with reduced NF-kB/NLRP3 signaling in lung tissues. Overall, PEC attenuated LPS-induced lung inflammation and injury, accompanied by reduced oxidative stress and modulation of HDAC3/NF-κB/NLRP3 signaling. These findings support the potential of PEC as a preventive agent against excessive pulmonary inflammation.

## 1. Introduction

Acute lung injury (ALI), a life-threatening condition that often leads to respiratory failure and severe pulmonary inflammation, is characterized by rapid onset, alveolar epithelial damage, protein-rich alveolar edema, and an uncontrolled inflammatory response driven by excessive immune cell infiltration [1]. Oxidative stress and dysregulated cell death pathways further exacerbate the pulmonary inflammation and tissue injury, contributing to the high morbidity and mortality associated with ALI [2]. ALI can be triggered by diverse insults, including sepsis, pneumonia caused by bacterial, viral, or fungal infections, mechanical trauma, acute pancreatitis, and other systemic inflammatory stimuli [3,4]. Despite extensive research, there are currently no approved pharmacological therapies that specifically target the underlying pathogenic mechanisms of ALI or its more severe form, acute respiratory distress syndrome (ARDS). Therefore, there remains an urgent need to develop novel therapeutic strategies targeting the molecular pathways responsible for disease progression.

Several studies have shown that alveolar epithelial cells and macrophages play critical and interconnected roles in the pathogenesis of ALI. Alveolar epithelial cells are essential components of the alveolar–capillary barrier, contributing to both barrier integrity and immunological regulation. Injury to these cells, along with the subsequent disruption of epithelial barrier function, is a critical determinant in the initiation and progression of ALI [5,6]. Upon activation by lipopolysaccharide (LPS), macrophages act as key effector cells that drive inflammatory responses and tissue injury. Macrophage death through programmed cell death pathways, including pyroptosis and apoptosis, further amplifies lung injury [7]. Moreover, under pathological conditions, macrophages can contribute to both epithelial injury and epithelial repair, reflecting their dual roles in disease progression [8]. Current management of ALI primarily relies on supportive care, including lung protective ventilation, antimicrobial therapy when appropriate, and treatment of the underlying cause. However, effective pharmacological therapies specifically targeting the pathogenic mechanisms of ALI remain unavailable.

As a key regulator of inflammatory cytokine maturation, the NOD-like receptor family pyrin domain-containing protein 3 (NLRP3) inflammasome is a multi-protein intracellular inflammatory signaling pathway that plays an essential role in innate immune defense [9]. Further, activation of the NLRP3 inflammasome contributes to the development of ALI by inducing pyroptosis, a highly inflammatory form of programmed cell death characterized by the activation of cleaved caspase-1 and the pore-forming protein gasdermin D (GSDMD) [10,11]. Nuclear factor-kappa B (NF-κB), a transcription factor that regulates the expression of various pro-inflammatory cytokines, acts as an upstream activator of NLRP3 [12]. Therefore, dysregulation of the NF-κB/NLRP3 inflammasome in the lung can contribute to respiratory diseases, suggesting that the NF-κB/NLRP3 inflammasome is a potential target for treating respiratory disorders, including ALI.

Histone deacetylases (HDACs) are enzymes that deacetylate histones and non-histone proteins in the cytoplasm, nucleus, and mitochondria [13]. In recent years, the roles of HDACs in ALI have been gradually elucidated. Although HDAC3 has been reported to regulate diverse biological processes in a context-dependent biological processes, recent evidence indicates that it promotes inflammatory responses and pyroptosis during LPS-induced ALI [14]. Collectively, these findings suggest that HDAC3 may represent a promising therapeutic target for mitigating inflammation and ALI.

Flavonoids are naturally occurring phytochemicals widely found in fruits, vegetables, leaves, and other plant-derived sources and have been routinely consumed as part of the human diet for decades. Extensive research has demonstrated that flavonoids exert diverse biological and pharmacological activities, including antioxidant, anti-inflammatory, antiviral, and anticancer effects [15,16]. These benefits are largely attributed to their ability to modulate a variety of cellular signaling pathways, making flavonoids promising candidates for therapeutic development in multiple disease contexts. Pectolinarigenin (PEC) is a flavonoid compound isolated from several traditional medicinal herbs, such as *Linaria vulgaris* and *Cirsium* species. PEC has attracted increasing attention due to its anti-inflammatory and anticancer activities. Several studies have demonstrated that PEC suppresses inflammatory signaling pathways and oxidative stress in various experimental disease models. For instance, PEC exhibits anti-inflammatory effects by inhibiting cyclooxygenase-2 (COX-2)-mediated prostaglandin E2 production and 5-lipoxygenase-mediated leukotriene synthesis in LPS-treated RAW264.7 macrophages [17]. In addition, PEC suppresses the NF-κB/MAPK signaling pathway, thereby exerting anti-inflammatory and cytoprotective effects [18]. Although corticosteroids are widely used to suppress excessive inflammation in selected patients with ARDS, their clinical benefits remain controversial because of inconsistent therapeutic outcomes and concerns regarding adverse effects, including immunosuppression and secondary infections. Accordingly, naturally derived compounds with anti-inflammatory and antioxidant properties have attracted increasing interest as potential therapeutic candidates for inflammatory lung diseases. Unlike corticosteroids, which broadly suppress immune response, PEC has been reported to modulate multiple inflammatory and oxidative stress-related signaling pathways, suggesting its potential as a multi-target therapeutic candidate. Despite these promising biological activities, the potential therapeutic effects of PEC in LPS-induced ALI and underlying molecular mechanisms remain unclear.

Therefore, the present study aimed to evaluate the protective effects of PEC against LPS-induced lung inflammation and to investigate the involvement of the HDAC3/NF-κB/NLRP3 signaling pathway. Using in vitro models consisting of MLE12 lung epithelial cells and RAW264.7 macrophages, together with a prophylactic C57BL/6 mouse model, we evaluated the effects of PEC on inflammatory responses, oxidative stress, and pyroptosis induced by LPS.

## 2. Materials and Methods

### 2.1. Reagents

PEC (purity > 99.00%, #GN10183) was purchased from GlpBio Technology Inc. (Montclair, CA, USA). LPS from *Escherichia coli* (#O55:B5), 3-(4,5-dimethylthiazol-2-yl)-2,5-diphenyltetrazolium bromide (MTT), human transferrin, β-estradiol, hydrocortisone, sodium selenite, and Griess reagent were obtained from Sigma-Aldrich. Insulin solution was purchased from Welgene Inc. (Gyeongsan, Gyeongsangbuk-do, Republic of Korea). RGFP966 was obtained from MedChemExpress (Monmouth Junction, NJ, USA). Dulbecco’s Modified Eagle Medium (DMEM), DMEM/F12, fetal bovine serum (FBS), and penicillin/streptomycin were purchased from Gibco (Waltham, MA, USA). 2,7-Dichlorofluorescein-diacetate (DCF-DA) was purchased from Abcam (Cambridge, MA, USA). Radioimmunoprecipitation assay (RIPA) lysis buffer containing a protease/phosphatase inhibitor cocktail (#1861280) was obtained from Thermo Scientific (Waltham, MA, USA). The following primary antibodies were used: anti-NF-κB p65, anti-phospho-NF-κB p65, anti-NLRP3, anti-HDAC3, anti-GSDMD, anti-cleaved caspase-1, and anti-lamin B, all from Cell Signaling Technology (Danvers, MA, USA). Anti-IκBα, anti-phospho-IκBα, and anti-β-actin were purchased from Santa Cruz Biotechnology (Dallas, TX, USA). Anti-Nrf2, anti-caspase-1, anti-histone H3, and anti-acetyl-histone H3K27 were obtained from Abclonal (Woburn, MA, USA). Alexa Fluor 488-conjugated and Alexa Fluor 568-conjugated secondary antibodies (used at 1:1000), as well as Fluoromount-G Mounting Medium, were obtained from Invitrogen (Waltham, MA, USA).

### 2.2. Cell Culture

The murine RAW264.7 macrophage cell line and murine lung epithelial cell line MLE12 were obtained from the Korean Cell Line Bank (Seoul, Republic of Korea) and American Type Culture Collection (Manassas, VA, USA), respectively. RAW264.7 macrophages were cultured in DMEM with 10% FBS. MLE12 cells were maintained in DMEM/F12 with 2% FBS, 1% penicillin/streptomycin, insulin 0.005 mg/mL, transferrin 0.01 mg/mL, sodium selenite 30 nM, hydrocortisone 10 nM, β-estradiol 10 nM, and L-glutamine 2 mM. Both cell lines were maintained at 37 °C in a humidified atmosphere containing 5% CO_2_. RAW264.7 macrophages and MLE12 cells were pretreated with PEC for 4 h and subsequently stimulated with LPS at 100 ng/mL and 10 µg/mL, respectively, according to previously established experimental protocols [19,20], for the indicated assays.

### 2.3. Cell Viability Assay

Cells were incubated at 1 × 10^4^ cells/well in a 96-well plate and grown overnight, followed by treatment with the indicated concentrations of PEC (either alone or in combination with LPS) for 18 h. Thereafter, 100 μL of MTT solution (5 mg/mL) was added to each well and incubated for an additional 2 h at 37 °C. The supernatant was removed, and 200 μL of dimethyl sulfoxide (final concentration, 100% *v*/*v*) was added to dissolve the purple formazan crystals. Quantification was performed by measuring the absorbance at 540 nm using a microplate reader (Promega, Madison, WI, USA). The results are presented as the mean values from three independent experiments performed in triplicate. The half-maximal inhibitory concentration (IC_50_) values were calculated using dose–response curves and GraphPad Prism 10 (GraphPad Software, Inc., La Jolla, CA, USA). Three independent experiments were carried out in triplicate.

### 2.4. Measurement of Nitrite Levels

Nitric oxide (NO) production was estimated by measuring nitrite using the Griess reaction. A sodium nitrite (NaNO_2_) standard curve was used for quantification. Equal volumes of cell culture supernatant, bronchoalveolar lavage fluid (BALF), or standard solutions were mixed with an equal volume of 1× Griess reagent and incubated at 25 °C. The absorbance was measured at 540 nm using a microplate reader (Promega, Madison, WI, USA). Three independent biological experiments were performed.

### 2.5. Quantitative Real-Time Polymerase Chain Reaction (qRT-PCR)

Total RNA was extracted from RAW264.7 macrophages and MLE12 cells using the RNA Extraction Kit (#K-3141, Bioneer, Daejeon, Republic of Korea). To quantitatively evaluate the mRNA levels of target genes, 1 μg of total RNA was converted to cDNA using ReverTra AceTM qPCR RT Master Mix with gDNA Remover (#FSQ-301, Toyobo, Osaka, Japan), following the manufacturer’s protocols [21]. qRT-PCR analysis was performed using Power SYBRTM Green PCR Master Mix (#4368577, Thermo Fisher Scientific, Waltham, MA, USA) on the real-time PCR cycler QuantStudio3 (Thermo Fisher Scientific) using target gene-specific primers. Glyceraldehyde-3-phosphate dehydrogenase was used as the internal control, and relative gene expression levels were calculated using the 2^−∆∆^CT method. The results are presented as the ratio to the internal reference. The primers used in the present study are listed in Table 1. Three independent biological experiments were performed.

### 2.6. Measurement of Intracellular Reactive Oxygen Species (ROS)

Intracellular ROS levels were determined using 2′,7′-dichlorofluorescein diacetate (DCF-DA). Cells were pretreated with various concentrations of PEC, followed by stimulation with LPS. During the final 30 min of LPS stimulation, cells were incubated with 20 μM DCF-DA at 37 °C. After incubation, the cells were detached using 0.25% trypsin and analyzed by flow cytometry (Agilent NovoCyte, Santa Clara, CA, USA). Three independent biological experiments were performed.

### 2.7. Western Blot Analysis

Whole-cell lysates were prepared using RIPA lysis buffer containing a protease/phosphatase inhibitor cocktail on ice. Nuclear and cytosolic protein fractions were isolated using the Subcellular Protein Fractionation Kit (Thermo Fisher Scientific, Waltham, MA, USA) according to the manufacturer’s instructions. Equal amounts of protein (30 μg) from each sample were separated on 4–20% sodium dodecyl sulfate–polyacrylamide gels and transferred onto polyvinylidene difluoride (PVDF) membranes. The membranes were blocked with 5% skim milk for 1 h at room temperature and then incubated overnight at 4 °C with the indicated primary antibodies. Detailed information on the primary antibodies, including the catalog numbers, host species, RRIDs, and dilution factors, is provided Supplementary Table S1. All primary antibodies were used according to the manufacturers’ recommended conditions for Western blot analysis. After washing three times with Tris-buffered saline containing 0.1% Tween 20 (TBST), the membranes were incubated with HRP-conjugated secondary antibodies for 1 h at room temperature. Protein bands were visualized using Pierce ECL Western Blot substrate (Thermo Fisher Scientific) and imaged with an iBright Imaging System (Invitrogen). Band intensities were quantified using ImageJ (National Institutes of Health, Bethesda, MD, USA). Cytoplasmic proteins were normalized to β-actin, whereas nuclear proteins were normalized to Lamin B1. The purity of the nuclear and cytoplasmic fractions was verified using β-actin and Lamin B1 as cytoplasmic and nuclear marker proteins, respectively (Supplementary Figure S1).

### 2.8. Immunofluorescence Staining

Cells were pretreated with PEC for 4 h and subsequently stimulated with LPS for 18 h. The cells were washed with phosphate-buffered saline (PBS), fixed with 4% paraformaldehyde for 10 min at 37 °C, and permeabilized with PBS containing 0.1% TritonX-100. After blocking with 5% normal goat serum for 1 h at room temperature, the cells were incubated with the appropriate primary antibodies overnight at 4 °C. Cells were then washed and incubated with Alexa Fluor 488- or 568-conjugated secondary antibodies (1:500 dilution) for 1 h at room temperature. Nuclei were counterstained with 4′,6-diamidino-2-phenylindole (DAPI). Coverslips were mounted using Fluoromount-G Mounting Medium, and fluorescence images were acquired using a confocal microscope (Nikon, Tokyo, Japan) at 400 × magnification. Fluorescence intensity was quantified using ImageJ software (version 1.54p; National Institutes of Health, Bethesda, MD, USA). Three independent biological experiments were performed.

### 2.9. Murine Model of LPS-Induced Lung Inflammation

Male C57BL/6 mice (8 weeks old; DBL, Chungcheongbuk-do, Republic of Korea) were used for the study and maintained under standard housing conditions with a 12 h light/dark cycle. Animals were acclimatized for 1 week before experimentation and provided *ad libitum* access to food and water. Mice weighing approximately 25 g were randomly assigned to six groups (*n* = 5 mice per group): vehicle control, PEC alone (50 mg/kg), LPS + vehicle, LPS + PEC pretreatment (20 mg/kg), LPS + PEC pretreatment (50 mg/kg), and LPS + RGFP966 pretreatment (25 mg/kg). Vehicle, PEC (20 or 50 mg/kg), or RGFP966 (25 mg/kg) was administered intraperitoneally once daily for seven consecutive days before LPS challenge according to the assigned treatment group. PEC and RGFP966 were first dissolved in DMSO and subsequently diluted with PEG300, Tween-80, and sterile saline to obtain a final formulation containing 5% DMSO, 30% PEG300, 5% Tween-80, and 60% sterile saline (*v*/*v*/*v*/*v*). The formulations were sonicated and gently warmed at 60 °C until a homogeneous solution was obtained prior to intraperitoneal administration. The sample size (*n* = 5 mice per group) was selected based on our previous studies employing similar LPS-induced lung inflammation models and our previous experience with this established model [22,23]. The PEC doses (20 and 50 mg/kg) were selected based on previously published in vivo studies demonstrating the anti-inflammatory and pharmacological efficacy of PEC at comparable dose ranges in experimental disease models [24,25,26].

To establish the LPS-induced lung inflammation model, mice were anesthetized with isoflurane inhalation and placed in the supine position on an inclined platform. The tongue was gently extended using forceps, and LPS solution (2.5 mg/mL in sterile saline; 50 μL per mouse, corresponding to 125 μg/mouse [5 mg/kg body weight]) was administered by intratracheal instillation using a sterile yellow pipette tip. Successful intratracheal delivery was confirmed by the absence of reflux and maintenance of spontaneous respiration following the procedure. Mice were euthanized 24 h after LPS administration, and lung tissues were collected for subsequent analyses.

### 2.10. Histopathological Analysis

For histological analysis, the right lung lobes were trimmed and fixed in 10% neutral formalin for 24 h. Paraffin-embedded tissues were sectioned at 5 μm and stained with hematoxylin and eosin [27]. Histopathological evaluation was performed using five randomly selected, non-overlapping microscopic fields per animal at ×200 magnification. Lung injury scoring was independently conducted by two investigators blinded to the treatment groups. The lung injury score was calculated as the sum of the scores for alveolar wall thickening, neutrophil infiltration, intra-alveolar hemorrhage, and alveolar congestion. Each histopathological parameter was graded on a scale of 0–4 according to previously described methods [22,28].

### 2.11. Lung Wet-to-Dry (W/D) Ratio

The lung wet-to-dry (W/D) ratio was determined as an indicator of pulmonary edema. Twenty-four hours after LPS administrations, the left lung was excised and immediately weighed to determine the wet weight. The lung was then dried in an oven at 60 °C for 48 h and reweighed to obtain the dry weight. The W/D ratio was calculated as the ratio of wet weight to dry weight.

### 2.12. Harvesting of Lungs and BALF

Twenty-four hours after LPS administration, the left lung of each mouse was snap-frozen and homogenized for subsequent analyses. To collect BALF, mice were euthanized and their tracheas were immediately lavaged twice with 1 mL of ice-cold PBS via a catheter. The pooled BALF was centrifuged at 250× *g* for 10 min at 4 °C, and the resulting supernatants were aliquoted and stored at −80 °C for subsequent measurement of inflammatory cytokine enzyme-linked immunosorbent assay (ELISA), myeloperoxidase (MPO) activity, and nitric oxide (NO) quantification, and total protein quantification using the bicinchoninic acid (BCA) assay. BAL cells were used for total cell counting and neutrophil counting. Additionally, the BAL cells were transferred onto glass slides using a cytospin procedure (3000 rpm, 7 min), fixed with methanol, and stained with Hema-3 (Thermo Fisher Scientific) according to the manufacturer’s instructions. Cells were based on standard morphological and staining characteristics [29].

### 2.13. Enzyme-Linked Immunosorbent Assay (ELISA)

Cell culture supernatant and BALF were collected for cytokine analysis. Cytokine concentrations, including TNF-α, IL-1β, and IL-6 in BALF and IL-1β in cell culture supernatants, were quantified using commercially available ELISA kits (LABIS KOMA, Seoul, Republic of Korea) according to the manufacturer’s instructions.

### 2.14. Measurement of Myeloperoxidase (MPO) Activity

MPO activity was determined using a commercially available MPO assay kit (Abcam, Cambridge, UK) according to the manufacturers’ instructions. The absorbance was measured using a microplate reader (Promega, Madison, WI, USA), and MPO activity were calculated according to the manufacturer’s protocol.

### 2.15. Immunohistochemistry (IHC)

Paraffin-embedded lung tissues were sectioned at 5 μm. Following antigen retrieval, the sections were blocked with 5% bovine serum albumin (BSA) for 1h at room temperature and incubated overnight at 4 °C with primary antibodies against HDAC3, NF-κB p65, and GSDMD-N. After washing, the sections were incubated with the appropriate secondary antibodies for 1 h at room temperature. Immunoreactivity was visualized using 3,3’-diaminobenzidine (DAB; Cell Signaling Technology), and the sections were counterstained with hematoxylin. Representative images were acquired using a light microscope (Leica, Deerfield, IL, USA). Positive staining was quantified in five randomly selected fields per section using ImageJ software (version 1.54p; National Institutes of Health, Bethesda, MD, USA.

### 2.16. Statistical Analysis

Data are presented as the mean ± standard deviation (SD). All statistical analysis were performed using GraphPad Prism (Version 10; GraphPad Software, San Diego, CA, USA). The number of biological replicates for each experiment is indicated in the corresponding figure legends. Comparisons between two groups were analyzed using Welch’s *t*-test, whereas comparisons among multiple groups were analyzed using Welch’s one-way analysis of variance (ANOVA) followed by Dunnett’s T3 multiple-comparison test. Assumptions of normality and homogeneity of variance were evaluated prior to statistical analysis. Differences were considered statistically significant at *p* < 0.05.

## 3. Results

### 3.1. PEC Reduces LPS-Induced Oxidative Stress in Both RAW264.7 Macrophages and MLE12 Cells

**Figure 1:**
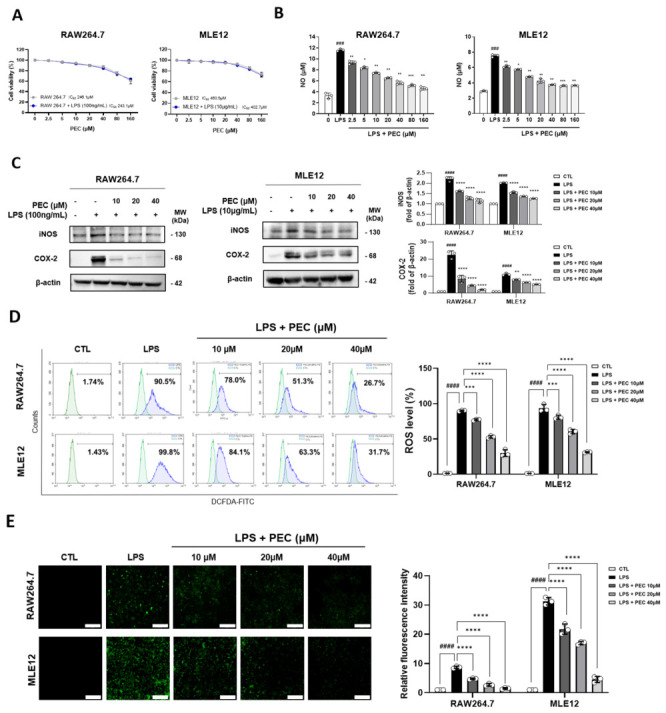
PEC attenuates LPS-induced oxidative stress in RAW264.7 macrophages and MLE12 cells. (**A**) Cell viability was measured using the MTT assay after pretreatment with PEC at the indicated concentrations for 4 h, followed by treatment with LPS in the presence or absence of PEC for an additional 18 h. (**B**–**E**) Cells were treated with LPS, PEC, or both for 18 h. When used in combination, cells were pretreated with PEC for 4 h before LPS stimulation. (**B**) Nitrite levels in the supernatants were assessed using a Griess assay. (**C**) Protein expression of iNOS and COX2 were analyzed using Western blot. Band intensities were quantified by densitometric analysis and normalized to β-actin. β-actin was used as the internal loading control. (**D**) The dose-dependent effects of PEC on cellular oxidative stress were evaluated by flow cytometry. The x-axis represents intracellular DCF-DA fluorescence intensity, and the y-axis indicates the number of cells. (**E**) Following treatment with LPS and/or PEC, the cells were washed and incubated with the H_2_DCF-DA probe. Representative fluorescence images of intracellular ROS levels are shown. Scale bar, 100 μm. Fluorescence intensity was quantified in five randomly selected fields per sample using the ImageJ software. Data are presented as the mean ± SD. All experiments were performed using at least three independent biological replicates, and representative data are shown. Compared with the control group: ### *p* < 0.001 and #### *p* < 0.0001; Compared with the LPS group, * *p* < 0.05, ** *p* < 0.01, *** *p* < 0.001 and **** *p* < 0.0001; COX-2, cyclooxygenase-2; DCF-DA, 2,7-dichlorofluorescein-diacetate; LPS, lipopolysaccharide; MTT, 3-(4,5-dimethylthiazol-2-yl)-2,5-diphenyltetrazolium bromide; PEC, pectolinarigenin; ROS, reactive oxygen species.

The effects of PEC on LPS-induced inflammatory responses were examined in RAW264.7 macrophages and MLE12 cells in vitro. First, we performed the MTT assay to evaluate the effects of PEC on cell viability. The IC_50_ values of PEC were 246.1 and 243.1 µM in RAW 264.7 macrophages treated with PEC alone or in combination with LPS, respectively, and 460.5 and 402.7 µM in MLE12 cells treated with PEC alone or in combination with LPS, respectively (Figure 1A). PEC showed minimal cytotoxicity at concentrations up to 40 µM after 18 h of treatments. iNOS-mediated NO production is a key indicator of inflammatory activation [30]. Therefore, nitrite levels, a stable end-product of NO, were measured to assess inflammatory responses. Nitrite levels were significantly increased in the LPS-treated group compared with those in the control group but were markedly reduced by PEC in RAW264.7 macrophages and MLE12 cells (Figure 1B). To further investigate this effect, iNOS and COX2 protein expression was examined by immunoblotting. LPS markedly increased the expression of iNOS and COX2, whereas PEC reduced their expression in a concentration-dependent manner in RAW264.7 macrophages and MLE12 cells (Figure 1C). Excessive ROS production is strongly associated with inflammatory lung injury and contributes to tissue damage [31]. Therefore, we measured intracellular ROS levels using DCF-DA staining followed by flow cytometry and fluorescence microscopy. LPS markedly increased ROS accumulation in both cell types, whereas PEC significantly attenuated intracellular ROS levels (Figure 1D–E). Cell-free control experiments demonstrated that PEC did not significantly interfere with the MTT, Griess, or DCF-DA assays (Supplementary Figure S2).

### 3.2. PEC Attenuates LPS-Induced NF-κB Activation and Inflammatory Response in RAW264.7 Macrophages and MLE12 Cells

**Figure 2:**
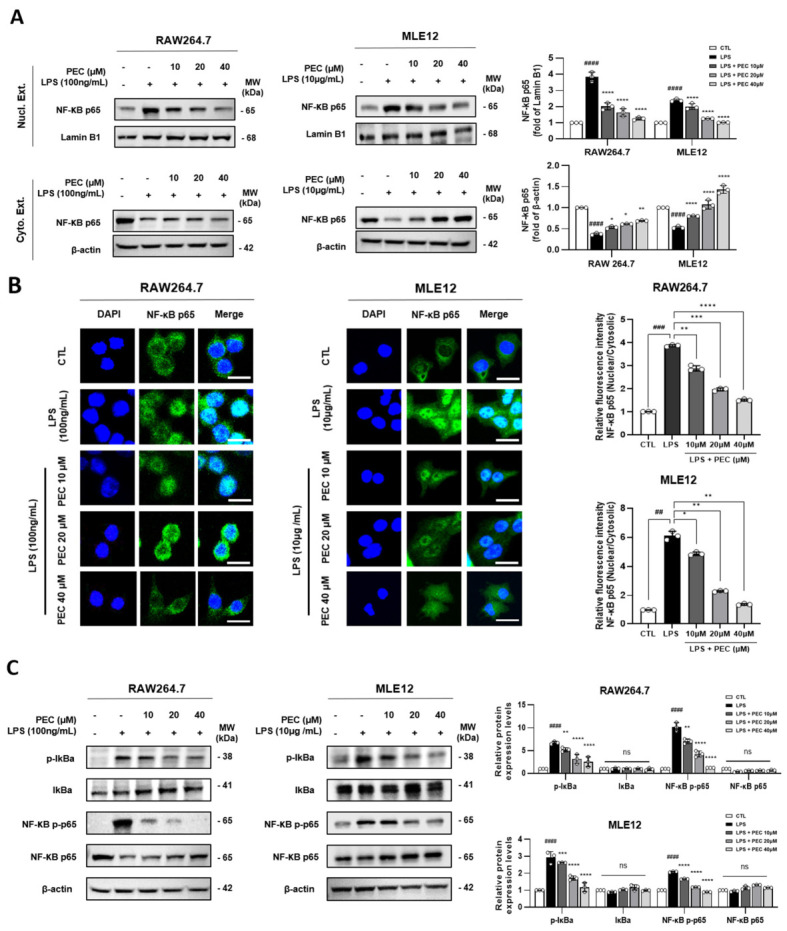
PEC attenuates LPS-induced NF-κB activation in RAW264.7 macrophages and MLE12 cells. (**A**–**C**) Cells were treated with LPS, PEC, or both for 18 h. When used in combination, cells were pretreated with PEC for 4 h before LPS stimulation. (**A**) Nuclear and cytoplasmic fractions were prepared, and NF-κB p65 nuclear translocation was analyzed by Western blot. Lamin B1 and β-actin were used as nuclear and cytoplasmic loading controls, respectively. Band intensities were quantified using ImageJ software. (**B**) Immunofluorescence staining of NF-κB p65 was performed, and nuclei were counterstained with DAPI. Scale bar, 10 μm. Relative fluorescence intensity was quantified in five randomly selected fields per sample using the ImageJ software. (**C**) Phosphorylation and protein expression of IκB-α and NF-κB p65 were analyzed by Western blot. Band intensities were quantified by densitometric analysis and normalized to β-actin. Data are presented as mean ± SD. All experiments were performed using at least three independent biological replicates, and representative data are shown. Compared with the control group: ## *p* < 0.01, ### *p* < 0.001 and #### *p* < 0.0001; Compared with the LPS group: * *p* < 0.05, ** *p* < 0.01, *** *p* < 0.001 and **** *p* < 0.0001; ns, not significant. DAPI, 4′,6′-diamidino-2-phenylindole; LPS, lipopolysaccharide; NF-κB, nuclear factor-kappa B; PEC, pectolinarigenin.

Aberrant activation of the NF-κB signaling pathway plays a major role in the development of LPS-induced lung inflammation [32]. Therefore, we examined NF-κB activation in LPS-stimulated RAW264.7 macrophages and MLE12 cells. Western blot analysis revealed that LPS stimulation markedly promoted the nuclear translocation of NF-κB in both cell lines, whereas PEC treatment reduced NF-κB nuclear translocation in a dose-dependent manner (Figure 2A). Consistently, immunofluorescence analysis showed that LPS stimulation induced an approximately two- to five-fold increase in NF-κB nuclear translocation in both cell lines, whereas treatment with PEC (10–40 μM) markedly attenuated this response (Figure 2B). We next examined the effect of PEC on NF-κB signaling and found that PEC reduced LPS-induced phosphorylation of IκB-α and p65 in RAW 264.7 macrophages and MLE12 cells (Figure 2C).

### 3.3. PEC Attenuates Pyroptosis-Associated Responses in RAW264.7 Macrophages and MLE12 Cells

**Figure 3:**
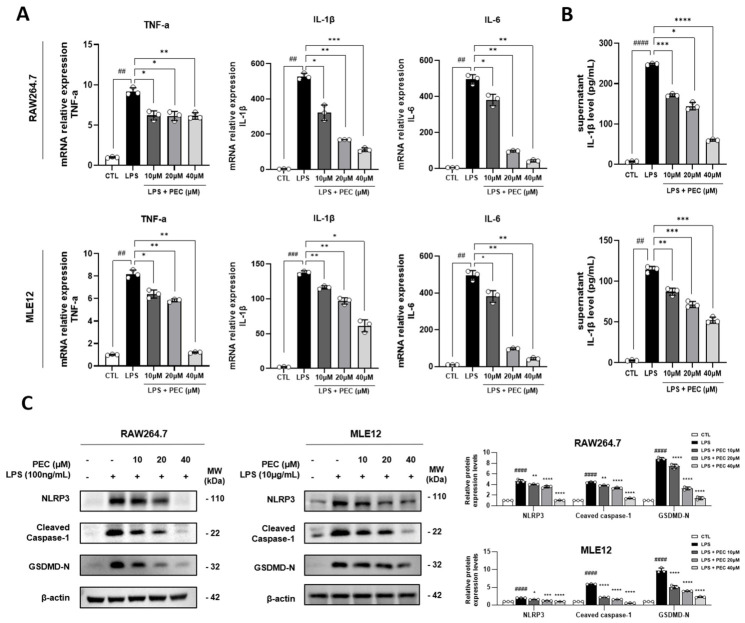
PEC reduced inflammatory cytokine production and NLRP3 inflammasome-associated signaling in RAW264.7 macrophages and MLE12 cells. (**A**) mRNA expression of TNF-α, IL-1β, and IL-6 were determined by qRT-PCR. (**B**) IL-1β secretion into the cell culture supernatants was quantified by ELISA. (**C**) Protein expression of NLRP3, cleaved caspase-1, and GSDMD-N were analyzed by Western blot. Band intensities were quantified using ImageJ software and normalized to β-actin. Data are presented as the mean ± SD. All experiments were performed using at least three independent biological replicates, and representative data are shown. Compared with the control group: ## *p* < 0.01, ### *p* < 0.001 and #### *p* < 0.0001; Compared with the LPS group: * *p* < 0.05, ** *p* < 0.01, *** *p* < 0.001 and **** *p* < 0.0001; GSDMD, gasdermin D; NLRP3, NOD-like receptor family pyrin domain-containing protein 3; PEC, pectolinarigenin; RT-PCR, real-time polymerase chain reaction.

PEC has been reported to exert antioxidant and anti-inflammatory activity in various inflammatory models [33]. Therefore, we further investigated the effects of PEC on LPS-induced inflammatory responses in RAW264.7 macrophages and MLE12 cells. Consistent with the mRNA expression data, LPS markedly increased the expression of TNF-α, IL-1β, and IL-6, whereas PEC significantly reduced their expression in both cell lines (Figure 3A). In addition, ELISA analysis demonstrated that LPS significantly increased IL-1β secretion into the culture supernatants, which was markedly attenuated by PEC pretreatment (Figure 3B). Having shown that PEC pretreatment attenuated LPS-induced oxidative stress (Figure 1D,E), we next examined whether these effects were associated with changes in NLRP3 inflammasome signaling. Western blot analysis revealed that LPS stimulation markedly increased the expression of NLRP3, cleaved caspase-1, and GSDMD-N in both RAW264.7 macrophages and MLE12 cells whereas PEC pretreatment dose-dependently reduced the expression of these pyroptosis-associated proteins (Figure 3C). Collectively, these results suggest that PEC suppresses LPS-induced inflammation activation and attenuates pyroptosis-associated inflammatory responses in RAW264.7 macrophages and MLE12 cells.

### 3.4. PEC Attenuates LPS-Induced Nuclear Accumulation of HDAC3 in RAW264.7 Macrophages and MLE12 Cells

**Figure 4:**
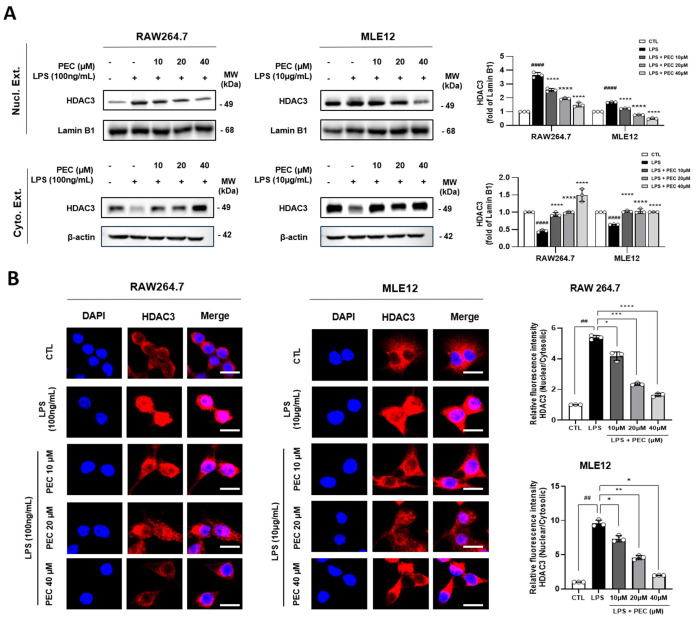
PEC reduces LPS-induced nuclear HDAC3 accumulation in RAW264.7 macrophages and MLE12 cells. (**A**) Nuclear and cytoplasmic protein fractions were isolated, and nuclear HDAC3 expression was analyzed by Western blot. Lamin B1 and β-actin were used as nuclear and cytoplasmic loading controls, respectively. Band intensities were quantified using ImageJ software (**B**) The nuclear localization of HDAC3 was detected via immunofluorescence, and nuclei were counterstained with DAPI. Scale bar, 10 μm. Relative fluorescence intensity was measured in five randomly selected fields per sample using the ImageJ software. Data are presented as the mean ± SD. All experiments were performed using at least three independent biological replicates, and representative data are shown. Compared with the control group ## *p* < 0.01, and #### *p* < 0.0001; Compared with the LPS group, * *p* < 0.05, ** *p* < 0.01, *** *p* < 0.001 and **** *p* < 0.0001; DAPI, 4′,6′-diamidino-2-phenylindole; HDAC, histone deacetylase; PEC, pectolinarigenin.

HDAC3 is increasingly recognized as an important regulator of inflammatory signaling, epithelial integrity, and pyroptosis in ALI, and accumulating evidence suggests that altered HDAC3 activity contributes to inflammatory lung injury [34,35]. Therefore, we first examined the effects of PEC on HDAC3 expression in LPS-stimulated RAW264.7 macrophages and MLE12 cells. Western blot analysis of nuclear and cytosolic fractions demonstrated that LPS stimulation markedly increased the nuclear HDAC3 levels in both cell types, whereas PEC treatment dose-dependently reduced its nuclear accumulation (Figure 4A). Consistent with the Western blot results, immunofluorescence analysis demonstrated increased nuclear localization of HDAC3 following LPS stimulation in RAW264.7 macrophages and MLE12 cells, whereas PEC treatment dose-dependently attenuated its nuclear localization (Figure 4B). These findings further support the inhibitory effect of PEC on LPS-induced HDAC3 nuclear translocation.

### 3.5. PEC Reduces NLRP3/Pyroptosis-Associated Signaling and RestoresH3K27 Acetylation in LPS-Treated RAW264.7 Macrophages and MLE12 Cells

**Figure 5:**
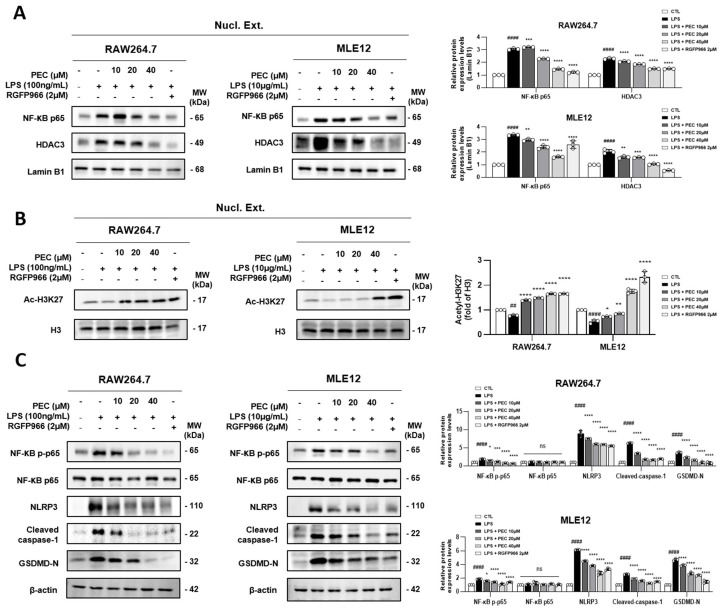
PEC restores H3K27 acetylation and reduces NF-κB/NLRP3-associated pyroptosis signaling in RAW264.7 macrophages and MLE12 cells. Cells were treated with LPS, PEC, or both for 18 h. When used in combination, cells were pretreated with PEC for 4 h before LPS stimulation. RGFP966, a selective HDAC3 inhibitor, was included as a pharmacological reference. (**A**) Nuclear protein fractions were isolated, and nuclear HDAC3 and NF-κB p65 levels were analyzed by Western blot. Lamin B1 was used as a nuclear loading control. (**B**) H3K27ac expression was analyzed by Western blot. H3 was used as a loading control for H3K27ac. (**C**) Protein expression of NF-κB p-p65, NLRP3, cleaved caspase-1, and GSDMD-N was analyzed by Western blot. Band intensities were quantified using ImageJ software. Data are presented as the mean ± SD. All experiments were performed using at least three independent biological replicates, and representative data are shown. Compared with the control group: ## *p* < 0.01 and #### *p* < 0.0001; Compared with the LPS group: * *p* < 0.05, ** *p* < 0.01, *** *p* < 0.001 and **** *p* < 0.0001; ns, not significant; GSDMD, gasdermin D; H3K27ac, histone H3 lysine 27 acetylation; HDAC, histone deacetylase; LPS, lipopolysaccharide; NF-κB, nuclear factor-kappa B; NLRP3, NOD-like receptor family pyrin domain-containing protein 3; PEC, pectolinarigenin.

To further examine the relationship between HDAC3-associated signaling and the anti-inflammatory effects of PEC, the selective HDAC3 inhibitor RGFP966 was included as a pharmacological comparator. Western blot analysis of nuclear fractions revealed that LPS stimulation markedly increased the nuclear accumulation of HDAC3 and NF-κB p65 in both cell lines, indicating enhanced inflammatory signaling. PEC pretreatment reduced the nuclear accumulation of HDAC3 and NF-κB p65 in a dose-dependent manner, producing effects comparable to those observed with RGFP966 treatment (Figure 5A). Because HDAC3 is involved in the regulation of histone acetylation, we next examined histone H3 lysine 27 acetylation (H3K27ac). LPS significantly reduced H3K27 acetylation in both cell lines, whereas PEC pretreatment restored H3K27ac levels (Figure 5B). RGFP966 also increased H3K27 acetylation, and PEC produced a comparable response, suggesting that the observed changes in H3K27 acetylation were associated with reduced HDAC3 expression (Figure 5B). Given that HDAC3 suppresses macrophage oxidative stress and pyroptosis by upregulating H3K27 acetylation [36], we next examined pyroptosis-associated proteins. LPS stimulation significantly increased the expression of phosphorylated NF-κB p65, NLRP3, cleaved caspase-1, and GSDMD-N. PEC pretreatment (40 μM) reduced the expression of these proteins, and the overall response was comparable to that observed with RGFP966 (Figure 5C). Taken together, these results indicate that the protective effects of PEC are associated with reduced HDAC3 expression, restoration of H3K27 acetylation, and attenuation of NF-κB/NLRP3-associated signaling. However, the present findings do not establish a direct molecular interaction between PEC and HDAC3, and additional mechanistic studies will be required to clarify this relationship.

### 3.6. PEC Attenuates LPS-Induced Lung Inflammation and Injury in Mice

**Figure 6:**
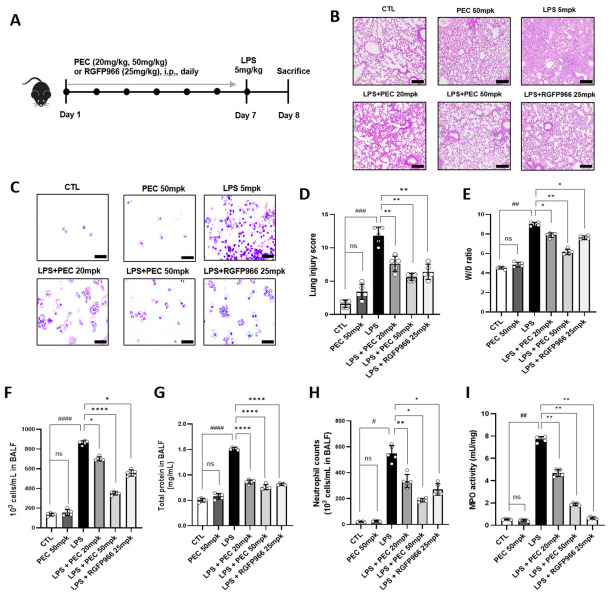
PEC attenuated LPS-induced lung inflammation and injury in mice. Mice were administered vehicle, PEC (20 or 50 mg/kg), or RGFP966 (25 mg/kg) via intraperitoneal injection once daily for 7 consecutive days (100 μL per injection). Following the final pretreatment, mice received an intratracheal instillation of LPS (5 mg/kg in 50 μL of sterile saline) or an equal volume of sterile saline. Twenty-four hours after LPS administration, mice were euthanized. Lung tissue, lung homogenates and BALF were collected for subsequent analyses. (**A**) Experimental scheme overview of the in vivo PEC and RGFP966 treatment protocol (*n* = 5 mice per group). (**B**) Representative H&E-stained lung sections from each experimental group. Scale bars, 200 μm. (**C**) BAL cells were stained with Giemsa staining and then observed under a microscope. Scale bars, 50 μm. (**D**) Quantification of lung injury scores. (**E**) Lung W/D weight ratio was used to assess pulmonary edema. (**F**) Total cell counts in BALF. (**G**) Total protein in BALF was measured using the BCA assay. (**H**) Neutrophil counts in BALF. (**I**) MPO activity in BALF. Data are presented as the mean ± SD (*n* = 5 per group). Compared with the CTL (vehicle control) group: # *p* < 0.05, ## *p* < 0.01, ### *p* < 0.001 and #### *p* < 0.0001; Compared with the LPS (vehicle-treated) group, * *p* < 0.05, ** *p* < 0.01 and **** *p* < 0.0001; ns, not significant; ALI, acute lung injury; BALF, bronchoalveolar lavage fluid; BCA, bicinchoninic Acid; H&E, hematoxylin and eosin; LPS, lipopolysaccharide; MPO, myeloperoxidase; PEC, pectolinarigenin; W/D, wet-to-dry.

Having shown that PEC reduced HDAC3 expression, attenuated NF-kB signaling, and decreased pyroptosis-associated response in vitro, we next examined its protective effects in vivo using an LPS-induced lung inflammation/injury mouse model. In this model, PEC or RGFP966 was administered intraperitoneally for seven consecutive days before, intratracheal LPS exposure on day 7. The experimental groups consisted of vehicle control, PEC alone (50 mg/kg), LPS + vehicle, LPS + PEC pretreatment (20 mg/kg), LPS + PEC pretreatment (50 mg/kg), and LPS + RGFP966 pretreatment (25 mg/kg) (Figure 6A). LPS challenge induced marked pulmonary inflammation characterized by inflammatory cell infiltration, alveolar septal thickening, and interstitial edema. PEC pretreatment at both 20 and 50 mg/kg markedly attenuated these histopathological changes in a dose-dependent manner, preserving alveolar architecture and reducing inflammatory cell infiltration (Figure 6B–D). Consistently, the lung wet-to-dry (W/D) ratio, an indicator of lung edema, was significantly increased in the LPS + vehicle group but was significantly reduced by PEC pretreatment (Figure 6E). Administration of PEC alone (50 mg/kg) produced no detectable histopathological alterations compared with the vehicle control group, indicating that PEC itself did not induce lung injury. RGFP966 produced protective effects comparable to those of PEC, whereas the greatest attenuation of lung injury was observed in the PEC 50 mg/kg group.

To further evaluate inflammatory cell infiltration, bronchoalveolar lavage fluid (BALF) was subjected to cytospin analysis. LPS challenge markedly increased the recruitment of macrophages and neutrophils, whereas PEC pretreatment significantly reduced inflammatory cell infiltration (Figure 6C). Consistent with these observations, both the total inflammatory cell counts and protein concentration in BALF were significantly increased in the LPS + vehicle group and were significantly reduced by PEC pretreatment (Figure 6F,G). Furthermore, quantitative analysis demonstrated that neutrophil counts in BALF were markedly elevated following LPS challenge but were significantly decreased by PEC pretreatment in a dose-dependent manner (Figure 6H). In addition, MPO activity in BALF was markedly elevated following LPS challenge but was significantly suppressed by PEC at both 20 and 50 mg/kg (Figure 6I). Collectively, these findings demonstrate that PEC pretreatment attenuated LPS-induced pulmonary inflammation and tissue injury, as evidenced by reduced inflammatory cell infiltration, pulmonary edema, neutrophil infiltration and MPO production in this prophylactic mouse model.

### 3.7. PEC Reduces Inflammatory Cytokine Production and iNOS Expression in LPS-Treated Mice

**Figure 7:**
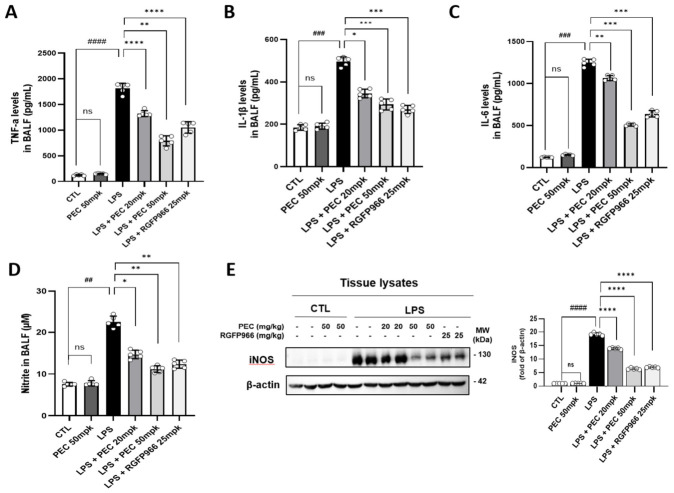
PEC reduced inflammatory cytokine production and iNOS expression in LPS-treated mice. Inflammatory cytokine levels in BALF, including (**A**) TNF-α, (**B**) IL-1β, and (**C**) IL-6, were measured by ELISA (*n* = 5 per group). (**D**) NO production was determined by measuring nitrite concentrations in BALF using the Griess reagent (*n* = 5 per group). (**E**) iNOS protein expression in lung tissues was analyzed by Western blot (*n* = 5 per group, and representative images are shown). Band intensities were quantified using ImageJ software and normalized to β-actin. Data are presented as mean ± SD. Compared with the CTL (vehicle control) group: ## *p* < 0.01, ### *p* < 0.001 and #### *p* < 0.0001; Compared with the LPS (vehicle-treated) group: * *p* < 0.05, ** *p* < 0.01, *** *p* < 0.001 and **** *p* < 0.0001; ns, not significant. BALF, bronchoalveolar lavage fluid; ELISA, enzyme-linked immunosorbent assay; IL, interleukin; iNOS, inducible nitric oxide synthase; LPS, lipopolysaccharide; NO, nitric oxide; PEC, pectolinarigenin; TNF-α, tumor necrosis factor- α.

To evaluate the anti-inflammatory effects of PEC in vivo, the levels of pro-inflammatory cytokine in BALF were determined. PEC pretreatment significantly reduced the LPS-induced increase in TNF-α, IL-1β, and IL-6 levels in BALF (Figure 7A–C). These findings indicate that PEC effectively attenuated the LPS-induced inflammatory response. Consistent with these observations, NO production, reflecting iNOS activity, was markedly increased in the BALF of LPS-treated mice but was significantly reduced by PEC pretreatment (Figure 7D). Furthermore, Western blot analysis demonstrated that LPS markedly increased iNOS protein expression in lung tissues, whereas PEC pretreatment dose-dependently suppressed this increase (Figure 7E). Together, these results demonstrate that PEC suppressed LPS-induced inflammatory cytokine production and iNOS expression in vivo.

### 3.8. PEC Reduces HDAC3 Expression and NF-κB/NLRP3-Associated Signaling in the Lung Tissue of LPS-Treated Mice

**Figure 8:**
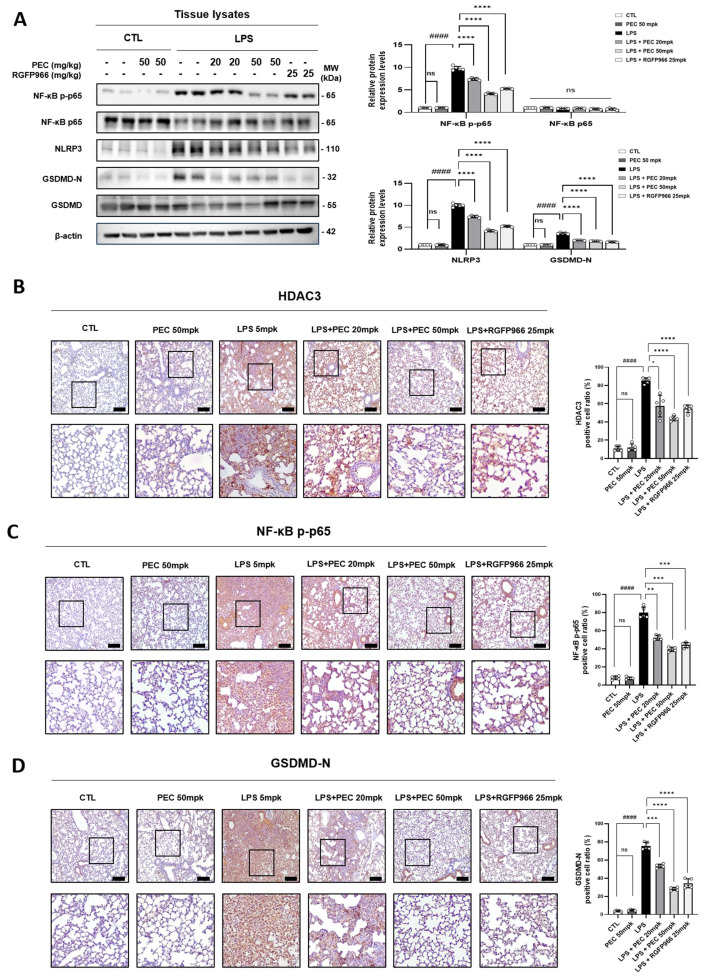
PEC attenuated HDAC3 expression and NF-κB/NLRP3-associated signaling in the lung tissues of LPS-treated mice. (**A**) Protein expression of NF-κB p-p65, NLRP3, and GSDMD-N in lung tissues was analyzed by Western blot. β-actin, NF-κB p65, and full-length GSDMD were used as loading controls. Band intensities were quantified by densitometric analysis. Immunohistochemical staining for (**B**) HDAC3, (**C**) NF-κB p-p65, and (**D**) GSDMD-N was performed (*n* = 5 per group, and representative images are shown). The boxed regions in the upper panels are shown at higher magnification in the corresponding lower panels. Quantification of positive cells was performed in five randomly selected fields per sample using ImageJ software. Dark brown staining indicates positive cells. Scale bars, 200 μm. Data are presented as mean ± SD. Compared with the CTL (vehicle control) group: #### *p* < 0.0001; Compared with the LPS (vehicle-treated) group: * *p* < 0.05, ** *p* < 0.01, *** *p* < 0.001 and **** *p* < 0.0001; ns, not significant; GSDMD, gasdermin D; HDAC3, histone deacetylase 3; NF-κB, nuclear factor kappa B; NLRP3, NOD-like receptor family pyrin domain-containing protein 3; PEC, pectolinarigenin; IHC, immunohistochemistry.

In vitro, PEC attenuated LPS-induced inflammatory responses and pyroptosis through the HDAC3/NF-κB/NLRP3 signaling. To determine whether these effects were also observed in vivo, we examined HDAC3 expression and pyroptosis-related signaling in lung tissues from LPS-treated mice (Figure 8A). LPS markedly increased NF-κB p65 phosphorylation, whereas PEC pretreatment dose-dependently reduced p65 phosphorylation, with the greatest inhibition observed at 50 mg/kg. Similarly, LPS increased the expression of the pyroptosis-related proteins NLRP3, and GSDMD-N, all of which were significantly reduced by PEC pretreatment in a dose-dependent manner. Immunohistochemical analysis further confirmed these findings, showing reduced HDAC3 expression, reduced NF-κB p65 phosphorylation, and diminished GSDMD-N staining in lungs of PEC-treated mice, with the most pronounced reduction observed in the 50 mg/kg group (Figure 8B–D). Collectively, these findings demonstrate that PEC attenuated HDAC3 expression and inhibited NF-κB/NLRP3-associated pyroptosis signaling in the lungs of LPS-treated mice.

## 4. Discussion

To date, most studies have highlighted the anti-inflammatory and modulatory effects of PEC in various disease models, including colitis, osteoarthritis, and several types of cancers [37,38,39]. These investigations collectively suggest that PEC exerts broad immunomodulatory activity. However, its protective effects against LPS-induced lung inflammation and the underlying molecular mechanisms have remained largely unexplored. In this study, PEC attenuated LPS-induced lung inflammation in both cellular models and a prophylactic mouse model. PEC treatment was associated with reduced pulmonary tissue injury, oxidative stress, and inflammatory responses, accompanied by modulation of the HDAC3/NF-κB/NLRP3 signaling pathway. These findings extend our understanding of the biological activity of PEC in experimental lung inflammation.

ALI, a life-threatening clinical syndrome with multifactorial origins, is characterized by uncontrolled pulmonary inflammation and disruption of the alveolar–capillary barrier, leading to progressive hypoxemia and respiratory failure [40]. Despite extensive research, there is currently no approved pharmacological therapy that specifically targets the underlying pathological mechanisms of ALI or ARDS, and current management remains largely supportive, focusing on treatment of the underlying causes, such as sepsis, pneumonia, aspiration, and trauma. Understanding the molecular mechanisms that drive inflammation and intervening in a timely manner are therefore critical for preventing the progression of lung injury. Experimentally, ALI can be induced by direct pulmonary exposure to noxious stimuli, including intratracheal or intranasal delivery of bacteria or bacterial components, such as LPS. LPS potently activates innate immunity and recruits inflammatory cells to the lungs, ultimately leading to the development of ALI [41,42]. In this study, we established an LPS-induced lung inflammation model to evaluate the protective effects of PEC.

Flavonoids are gaining recognition as protective agents against inflammatory lung injuries because of their pronounced antioxidative and anti-inflammatory effects [43,44]. Among them, PEC has garnered increasing attention as a natural flavonoid with well-documented regulatory effects on inflammatory and oxidative pathways. Prior reports indicate that PEC suppresses key inflammatory mediators while maintaining redox balance [10,38,45]. Our findings revealed that PEC substantially attenuated LPS-induced expression of TNF-α, IL-1β, and IL-6 in vitro and reduced the production of these pro-inflammatory cytokines in vivo (Figure 3A and Figure 7A–C). Furthermore, PEC significantly decreased IL-1β secretion into the culture supernatants (Figure 3B), supporting its inhibitory effect on inflammasome-associated inflammatory responses. Moreover, PEC significantly reduced oxidative stress, as evidenced by the downregulation of iNOS and COX-2 (Figure 1B,C and Figure 7D,E) as well as a marked reduction in DCF-DA-detected intracellular ROS (Figure 1D,E). These observations are consistent with previous reports showing that suppression of oxidative and nitrosative stress can alleviate inflammatory lung injury and further support the protective effects of PEC.

NF-κB is a transcription factor that plays an important role in physiological and pathological processes, including regulation of inflammatory responses and apoptosis [46]. Activation of multiple upstream receptors and signaling cascades converges on the NF-κB pathway, positioning it as a central node in the initiation and amplification of inflammatory signaling [47]. Because NF-κB controls the transcription of a broad range of pro-inflammatory cytokines, chemokines, and adhesion molecules, it is widely recognized as an important therapeutic target in inflammatory diseases. PEC pretreatment attenuated NF-kB activation, as evidenced by reduced phosphorylation of IκBα and p65, together with decreased production of TNF-α, IL-1β, and IL-6. These findings suggest that suppression of NF-κB signaling contributes, at least in part, to the anti-inflammatory effects of PEC during LPS-induced lung inflammation.

The NLRP3 inflammasome, a critical component of innate immunity, promotes the maturation and secretion of IL-1β through caspase-1 activation, ultimately leading to pyroptotic cell death [46,48]. Increasing evidence indicates that activation of the NLRP3 inflammasome and subsequent pyroptosis play a pivotal role in amplifying the severity of LPS-triggered pulmonary injury [49,50]. Although canonical NLRP3 inflammasome activation generally requires both a priming signal, previous studies have demonstrated that LPS alone can induce pyroptosisRAW264.7 macrophages and MLE12 cells under specific experimental conditions, as evidenced by increased PI-positive cells, LDH release, and GSDMD cleavage [19,20]. Therefore, we employed this established LPS-alone model to investigate the effects of PEC on LPS-induced inflammatory pyroptosis. Consistent with these mechanistic insights, prior studies have demonstrated that pharmacologic or genetic inhibition of NLRP3, caspase-1, or GSDMD-N markedly attenuates neutrophil accumulation, cytokine production, and histopathological damage in LPS-induced ALI models. [51,52]. Our findings showed that PEC effectively reduced NLRP3 and GSDMD-N expression in LPS-induced MLE12 epithelial cells, RAW264.7 macrophages (Figure 3B), and lung tissues (Figure 8A). These findings suggest that the protective effects of PEC are associated with reduced NF-κB/NLRP3 signaling and attenuation of pyroptosis-associated responses.

A key feature of ALI pathogenesis involves dysregulation of epigenetic mechanisms that orchestrate inflammatory gene transcription. HDAC3 has emerged as an important regulator linking chromatin remodeling to innate immune activation. HDAC3 is required for full induction of NF-κB-dependent inflammatory gene expression in response to stimuli [53,54,55,56]. Although PEC has previously been shown to regulate several signaling pathways, including NF-κB, JAK2/STAT3, Ras/ERK, and FGFR3 [38,39], its involvement in HDAC3-associated signaling has not been described. We found that PEC markedly suppressed the LPS-induced HDAC3 expression in a dose-dependent manner (Figure 4). Consistently, PEC reduced NF-κB activation and NLRP3 inflammasome markers, showing an inhibitory profile comparable to that of RGFP966, a selective HDAC3 inhibitor (Figure 5C). PEC also restored H3K27 acetylation in both RAW264.7 macrophages and MLE12 cells (Figure 5B). These observations suggest that reduced HDAC3 expression may contribute to the attenuation of downstream inflammatory signaling following PEC treatment. However, because direct mechanistic validation of HDAC3 was not performed, further studies are needed to clarify the relationship between PEC and HDAC3 signaling. Overall, PEC exerted protective effects against LPS-induced pulmonary inflammation in both cellular models and a prophylactic mouse model. These protective effects were accompanied by reduced pulmonary edema, inflammatory cell infiltration, pro-inflammatory cytokine production, oxidative stress, and pyroptosis-associated responses, together with decreased HDAC3 expression and reduced NF-κB/NLRP3 signaling. Although the present findings support the protective effects of PEC against excessive pulmonary inflammation, the study was performed using a prophylactic treatment protocol, and the direct regulatory relationship between PEC and HDAC3 remains to be clarified. Further studies using post-treatment models in both male and female mice, together with additional mechanistic investigations, are warranted to better define the role of HDAC3 signaling in the protective effects of PEC.

## 5. Conclusions

In conclusion, PEC attenuated LPS-induced inflammatory lung injury in both in vitro models and a prophylactic mouse model. PEC treatment was associated with reduced pulmonary edema, inflammatory cell infiltration, pro-inflammatory cytokine production, oxidative stress, and pyroptosis-associated signaling, together with decreased HDAC3 expression/nuclear localization and reduced NF-kB/NLRP3 signaling. To the best of our knowledge, this is the first study to demonstrate an association between PEC treatment and modulation of HDAC3/NF-kB/NLRP3 signaling in an experimental model of LPS-induced lung inflammation. Collectively, these findings suggest that PEC may represent a promising preventive candidate for LPS-induced inflammatory lung injury.

## Figures and Tables

**Table 1:** Sequences of gene-specific primers used for quantitative real-time PCR.

Gene	Species	Forward Primer (5′-3′)	Reverse Primer (3′-5′)
*TNF-α*	Mouse	CTTCTCATTCCTGCTTGTG	ACTTGGTGGTTTGCTACG
*IL-1β*	Mouse	TGGACCTTCCAGGATGAGGACA	GTTCATCTCGGAGCCTGTAGTG
*IL-6*	Mouse	TACCACTTCACAAGTCGGAGGC	CTGCAAGTGCATCATCGTTGTTC
*GAPDH*	Mouse	GGTTGTCTCCTGCGACTTCA	TGGTCCAGGGTTTCTTACTCC

## Data Availability

The data supporting the findings of this study are available from the corresponding author upon reasonable request. Full, uncropped Western blot images are provided in Supplementary File S1.

## Supplementary Materials

The following supporting information can be downloaded at: https://www.mdpi.com/article/10.3390/antiox15070898/s1, one supplementary table, two supplementary figures, and one supplementary file. Supplementary Table S1. Primary antibodies used for western blot analysis. Supplementary Figure S1. Validation of nuclear and cytoplasmic fractionation purity. Supplementary Figure S2. Evaluation of potential assay interference by pectolinarigenin (PEC) using cell-free control experiments. Supplementary File S1. Uncropped western blot images corresponding to Figure 1C, Figure 2A,C, Figure 3B, Figure 4A, Figure 5A–C, Figure 7E, and Figure 8A.

## Abbreviations

The following abbreviations are used in this manuscript:

ALI	Acute lung injury
BALF	Bronchoalveolar lavage fluid
COX-2	Cyclooxygenase-2
DCF-DA	Dichlorodihydrofluorescein diacetate
ELISA	Enzyme-linked immunosorbent assay
GSDMD	Gasdermin D
H&E	Hematoxylin and eosin
H3K27	Histone H3 at lysine 27
HDAC3	Histone deacetylase 3
IL-1β	Interleukin-1β
IL-6	Interleukin-6
iNOS	Inducible nitric oxide synthase
LPS	Lipopolysaccharide
MPO	Myeloperoxidase
MTT	3-(4,5-Dimethylthiazol-2-yl)-2,5-diphenyltetrazolium bromide
NF-κB	Nuclear factor-kappa B
NLRP3	NOD-like receptor family pyrin domain-containing 3
PEC	Pectolinarigenin
ROS	Reactive oxygen species
RT-qPCR	Reverse transcription quantitative polymerase chain reaction
TNF-α	Tumor necrosis factor-alpha
