# Supplementary material for: Pectolinarigenin Attenuates LPS-Induced Lung Inflammation and Injury with Reduced HDAC3/NF-κB/NLRP3 Signaling
Source: Antioxidants (Basel). 2026 Jul 20;15(7):898. doi: 10.3390/antiox15070898 (PMC13404758; doi:10.3390/antiox15070898)
Supplement: Supplementary file 1 [file antioxidants-15-00898-s001.zip › Supplementary File_S1.pdf]

Fig 1C.

RAW 2647

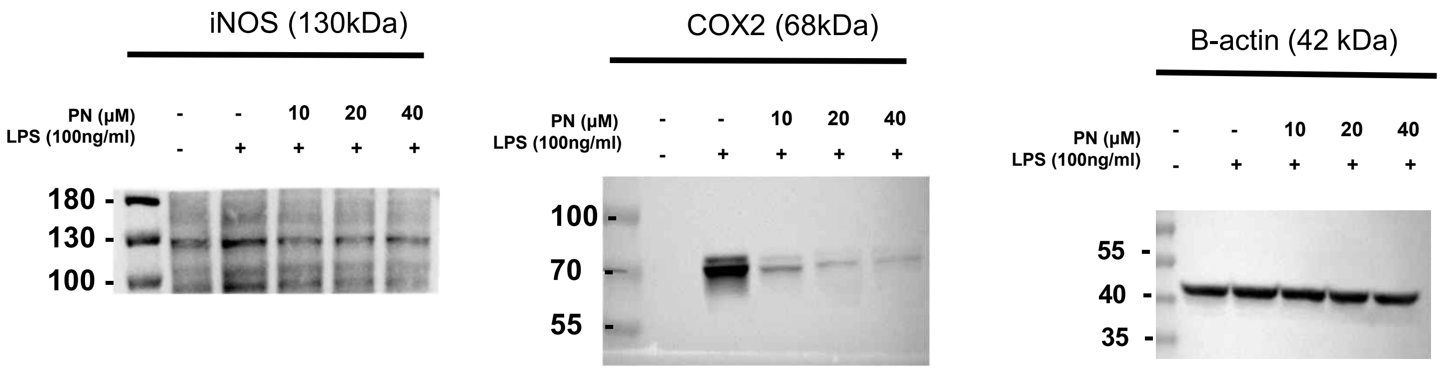

MLE12

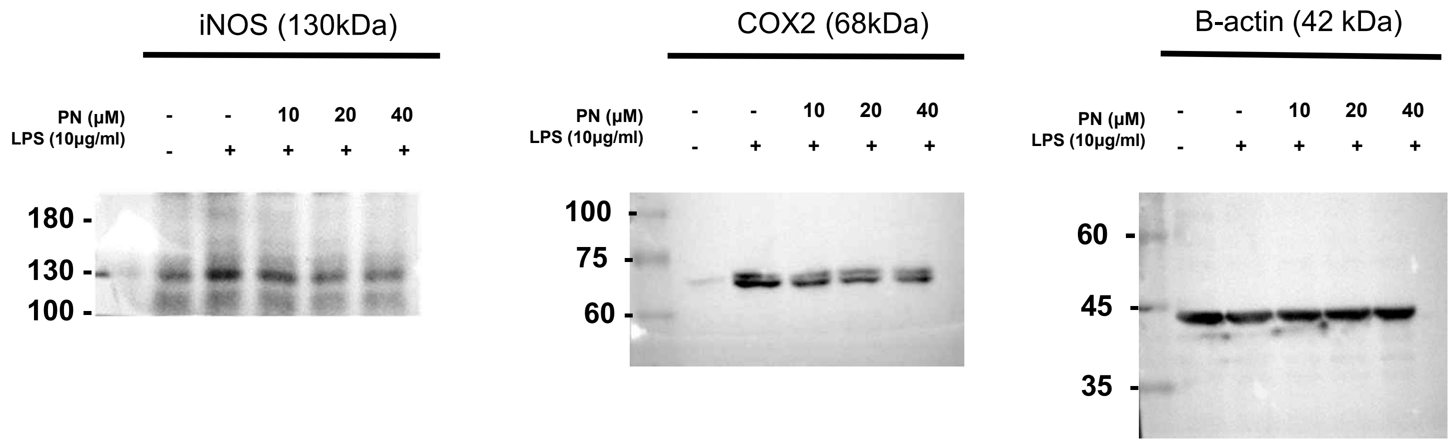

Fig 2A.

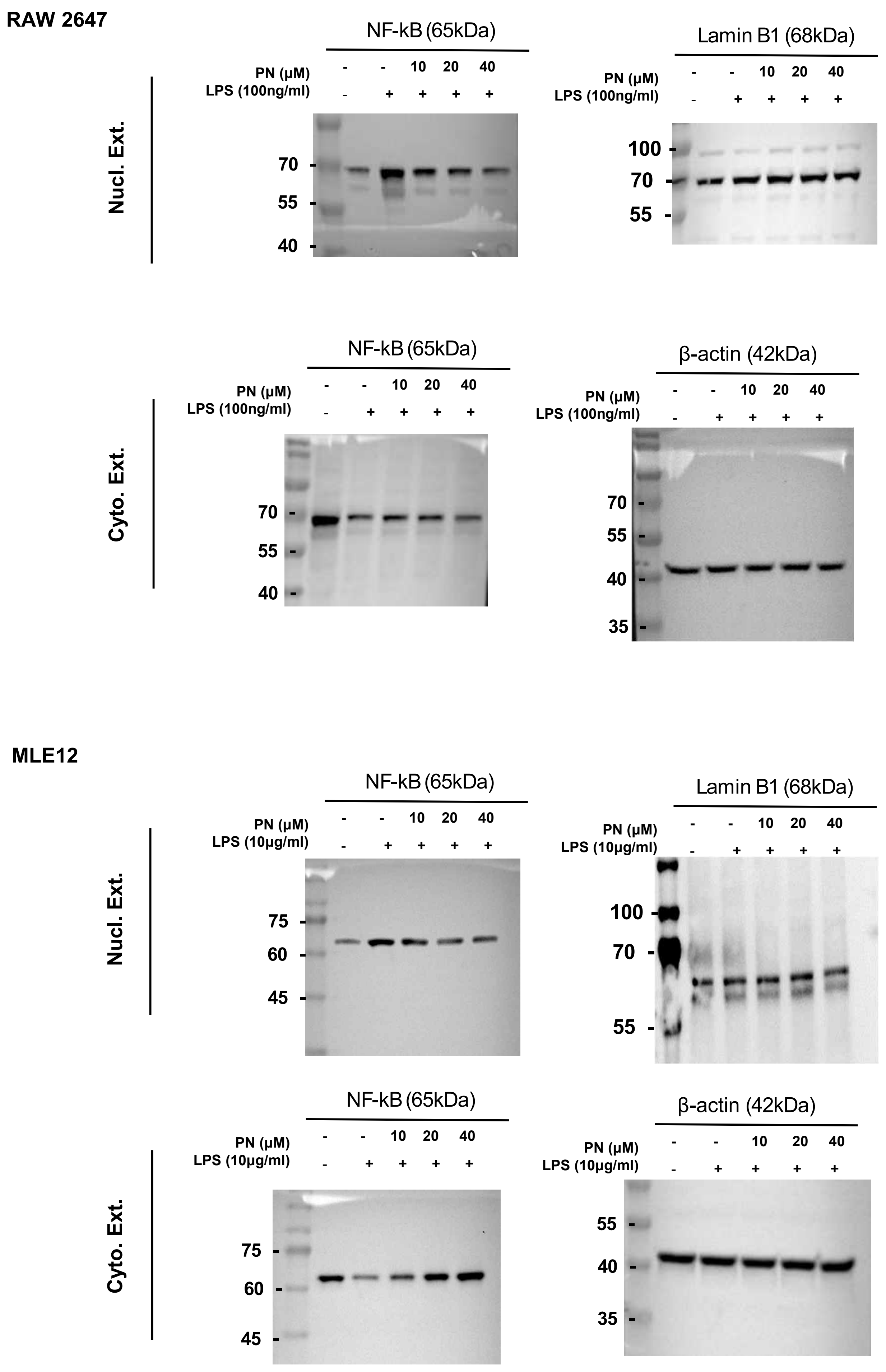
RAW 2647
NF-kB (65kDa)
Lamin B1 (68kDa)
PN (μM) - - 10 20 40
LPS (100ng/ml) - + + + +
Nucl. Ext.
70
55
40
100
70
55
NF-kB (65kDa)
β-actin (42kDa)
PN (μM) - - 10 20 40
LPS (100ng/ml) - + + + +
Cyto. Ext.
70
55
40
70
55
40
35
MLE12
NF-kB (65kDa)
Lamin B1 (68kDa)
PN (μM) - - 10 20 40
LPS (10μg/ml) - + + + +
Nucl. Ext.
75
60
45
100
70
55
NF-kB (65kDa)
β-actin (42kDa)
PN (μM) - - 10 20 40
LPS (10μg/ml) - + + + +
Cyto. Ext.
75
60
45
55
40
35

Fig.2C

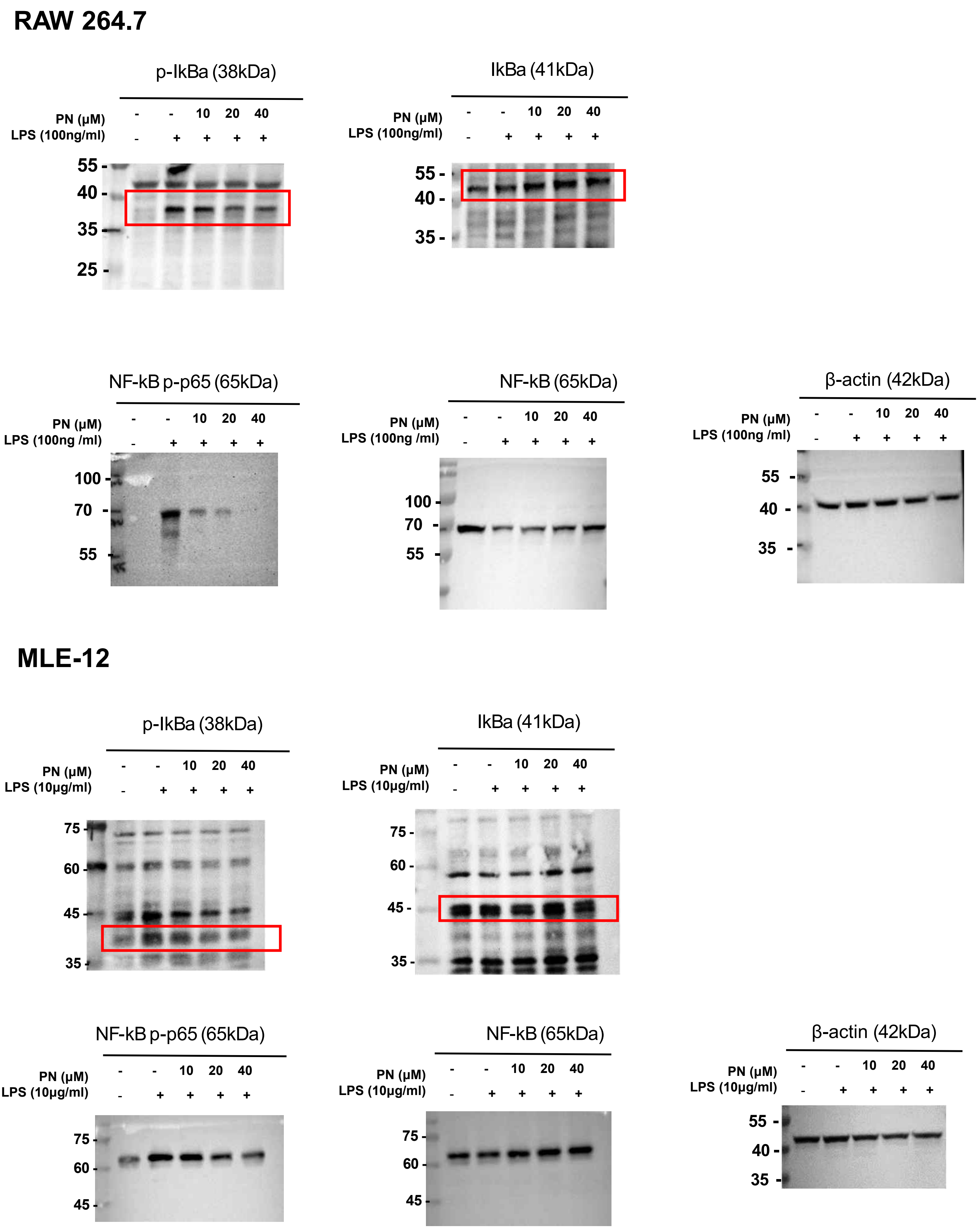
RAW 264.7
p-IkBa (38kDa)
PN (μM) - - 10 20 40
LPS (100ng/ml) - + + + +
55 40 35 25
IkBa (41kDa)
PN (μM) - - 10 20 40
LPS (100ng/ml) - + + + +
55 40 35
NF-kB p-p65 (65kDa)
PN (μM) - - 10 20 40
LPS (100ng /ml) - + + + +
100 70 55
NF-kB (65kDa)
PN (μM) - - 10 20 40
LPS (100ng /ml) - + + + +
100 70 55
β-actin (42kDa)
PN (μM) - - 10 20 40
LPS (100ng /ml) - + + + +
55 40 35
MLE-12
p-IkBa (38kDa)
PN (μM) - - 10 20 40
LPS (10μg/ml) - + + + +
75 60 45 35
IkBa (41kDa)
PN (μM) - - 10 20 40
LPS (10μg/ml) - + + + +
75 60 45 35
NF-kB p-p65 (65kDa)
PN (μM) - - 10 20 40
LPS (10μg/ml) - + + + +
75 60 45
NF-kB (65kDa)
PN (μM) - - 10 20 40
LPS (10μg/ml) - + + + +
75 60 45
β-actin (42kDa)
PN (μM) - - 10 20 40
LPS (10μg/ml) - + + + +
55 40 35

Fig.3B

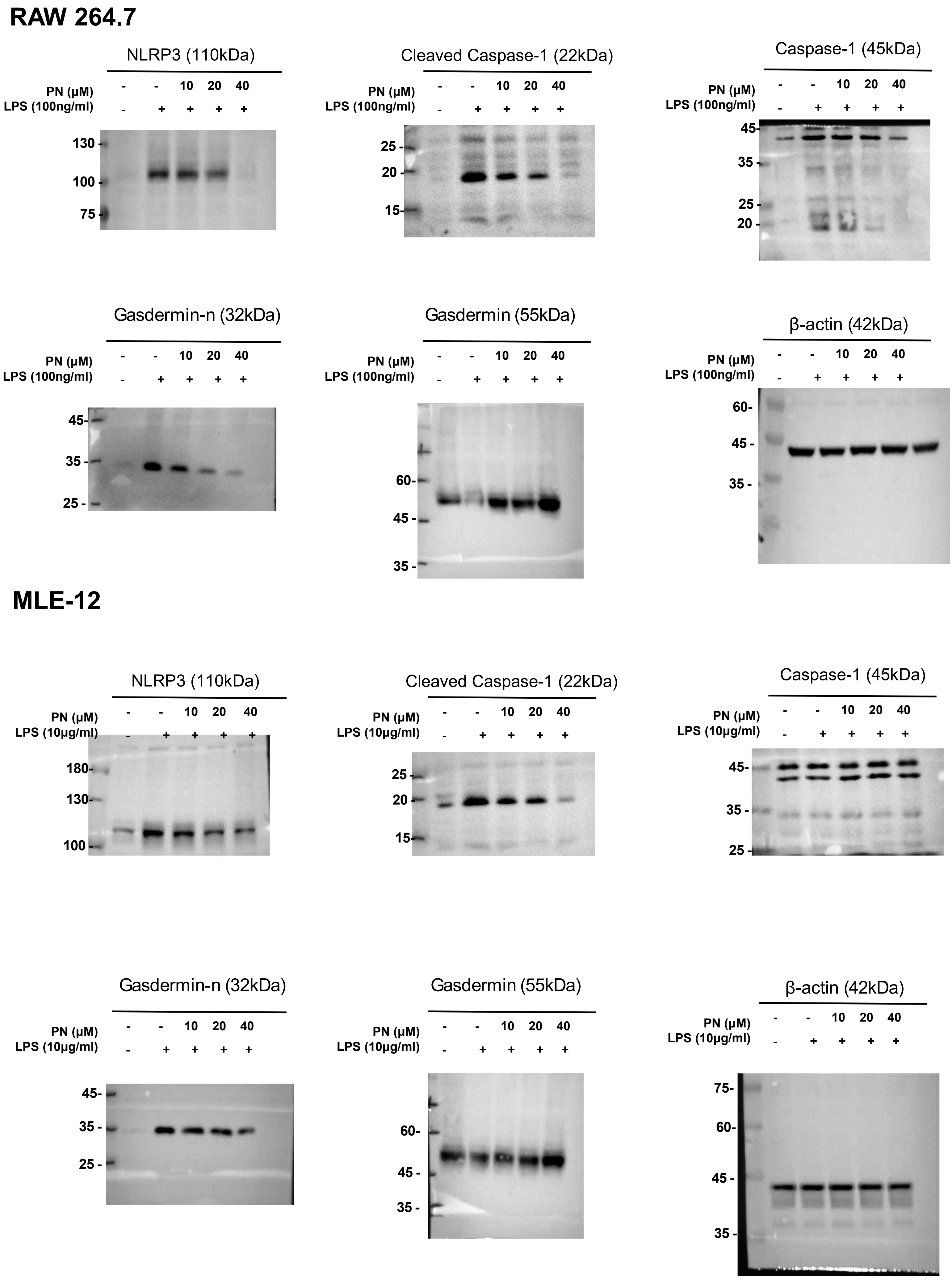
RAW 264.7
NLRP3 (110kDa)
PN (μM) - - 10 20 40
LPS (100ng/ml) - + + + +
130
100
75
Cleaved Caspase-1 (22kDa)
PN (μM) - - 10 20 40
LPS (100ng/ml) - + + + +
25
20
15
Caspase-1 (45kDa)
PN (μM) - - 10 20 40
LPS (100ng/ml) - + + + +
45
35
25
20
Gasdermin-n (32kDa)
PN (μM) - - 10 20 40
LPS (100ng/ml) - + + + +
45
35
25
Gasdermin (55kDa)
PN (μM) - - 10 20 40
LPS (100ng/ml) - + + + +
60
45
35
β-actin (42kDa)
PN (μM) - - 10 20 40
LPS (100ng/ml) - + + + +
60
45
35
MLE-12
NLRP3 (110kDa)
PN (μM) - - 10 20 40
LPS (10μg/ml) - + + + +
180
130
100
Cleaved Caspase-1 (22kDa)
PN (μM) - - 10 20 40
LPS (10μg/ml) - + + + +
25
20
15
Caspase-1 (45kDa)
PN (μM) - - 10 20 40
LPS (10μg/ml) - + + + +
45
35
25
Gasdermin-n (32kDa)
PN (μM) - - 10 20 40
LPS (10μg/ml) - + + + +
45
35
25
Gasdermin (55kDa)
PN (μM) - - 10 20 40
LPS (10μg/ml) - + + + +
60
45
35
β-actin (42kDa)
PN (μM) - - 10 20 40
LPS (10μg/ml) - + + + +
75
60
45
35

Fig.4A

RAW 264.7

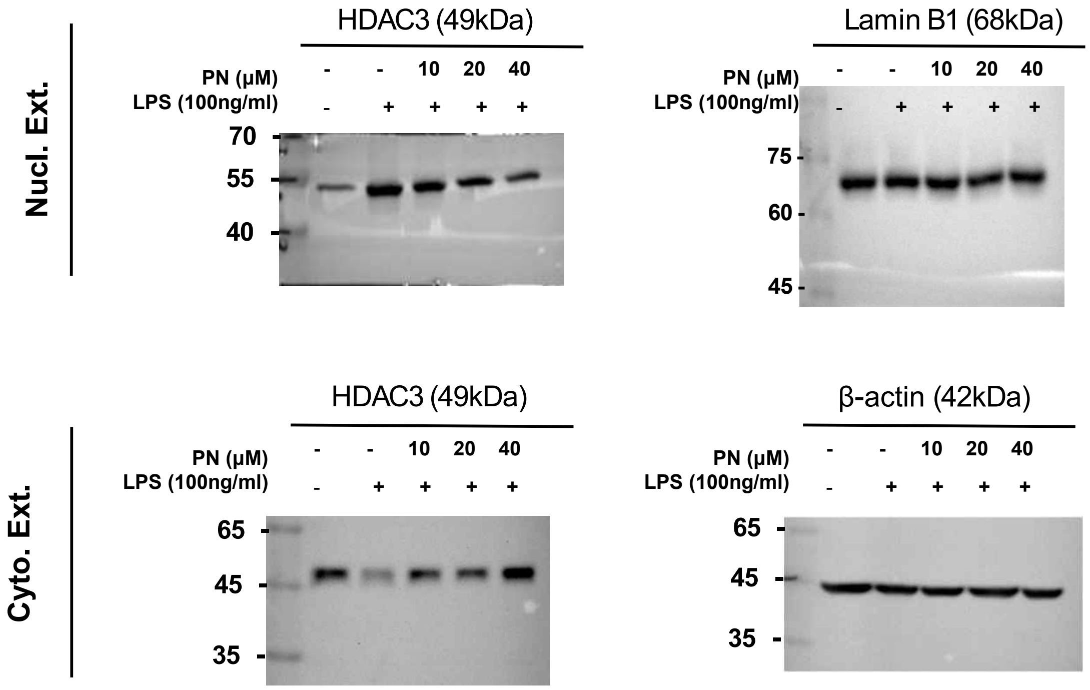

MLE-12

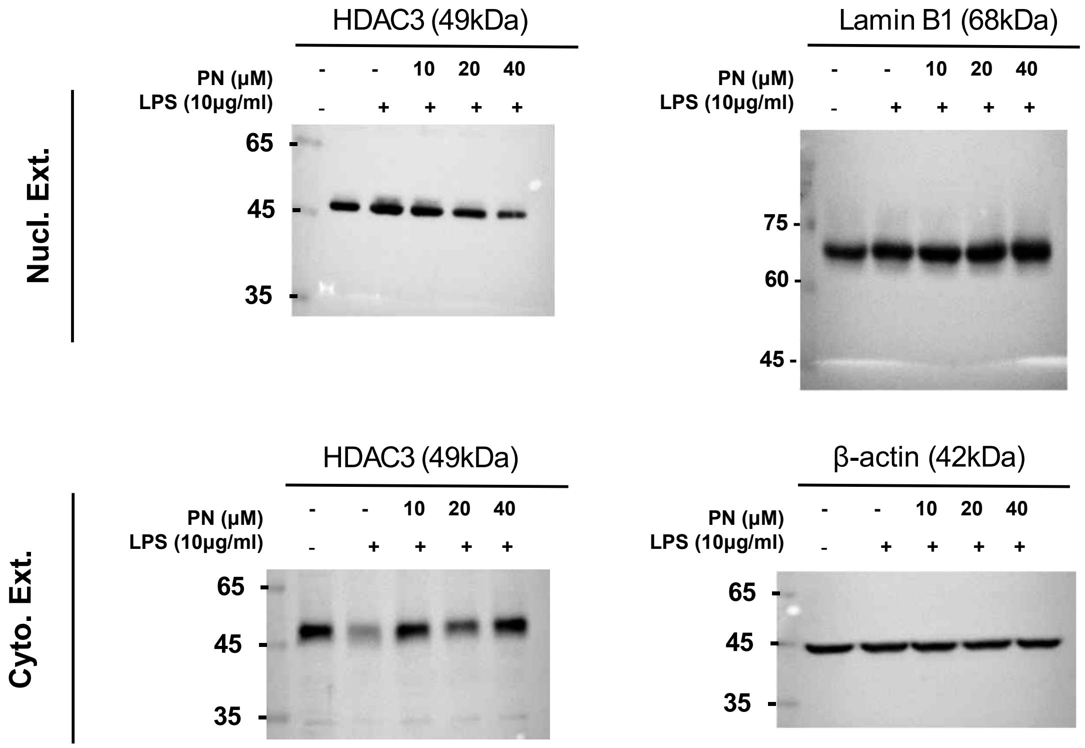

Fig.5A

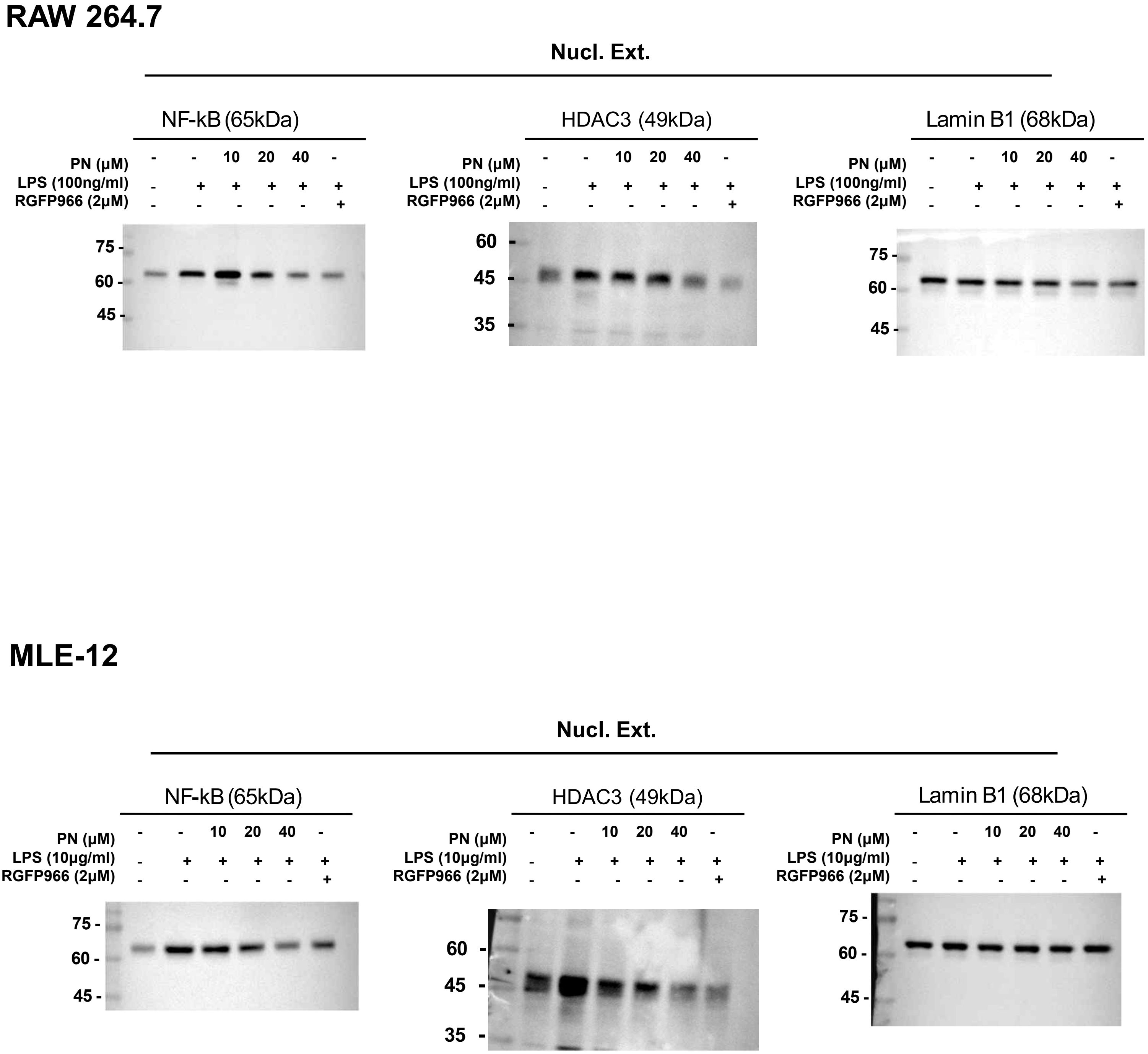
RAW 264.7
Nucl. Ext.
NF-kB (65kDa)
HDAC3 (49kDa)
Lamin B1 (68kDa)
PN (μM) - - 10 20 40 -
LPS (100ng/ml) - + + + + +
RGFP966 (2μM) - - - - - +
75
60
45
35
MLE-12
Nucl. Ext.
NF-kB (65kDa)
HDAC3 (49kDa)
Lamin B1 (68kDa)
PN (μM) - - 10 20 40 -
LPS (10μg/ml) - + + + + +
RGFP966 (2μM) - - - - - +

Fig.5B

RAW 264.7

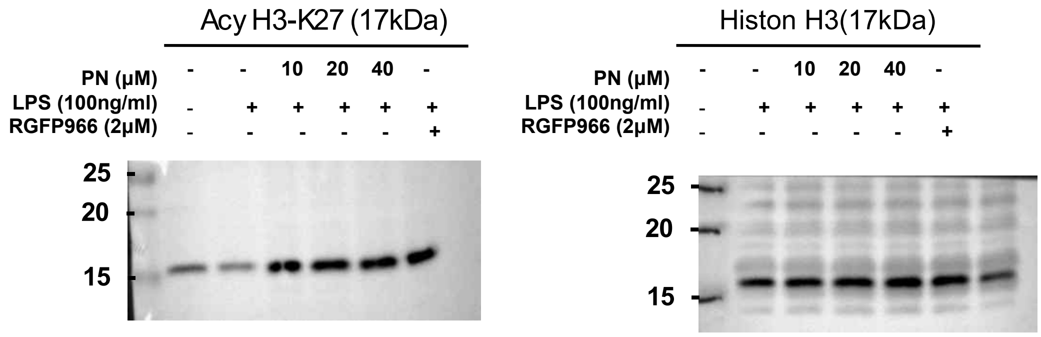

MLE-12

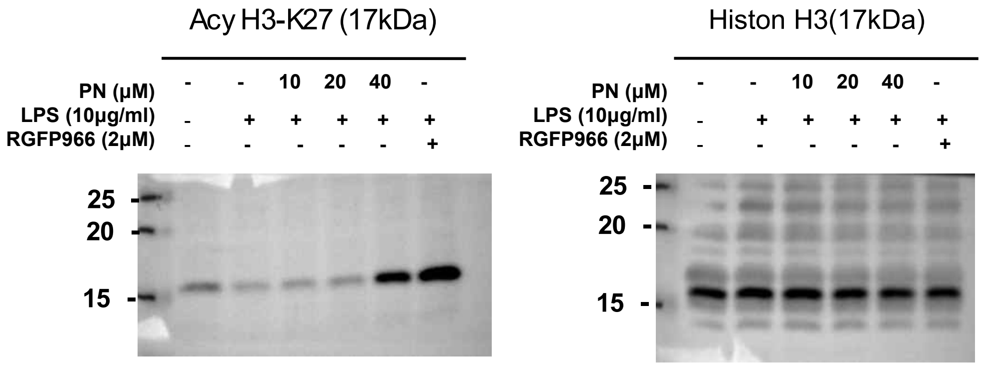

Fig.5C

RAW 264.7

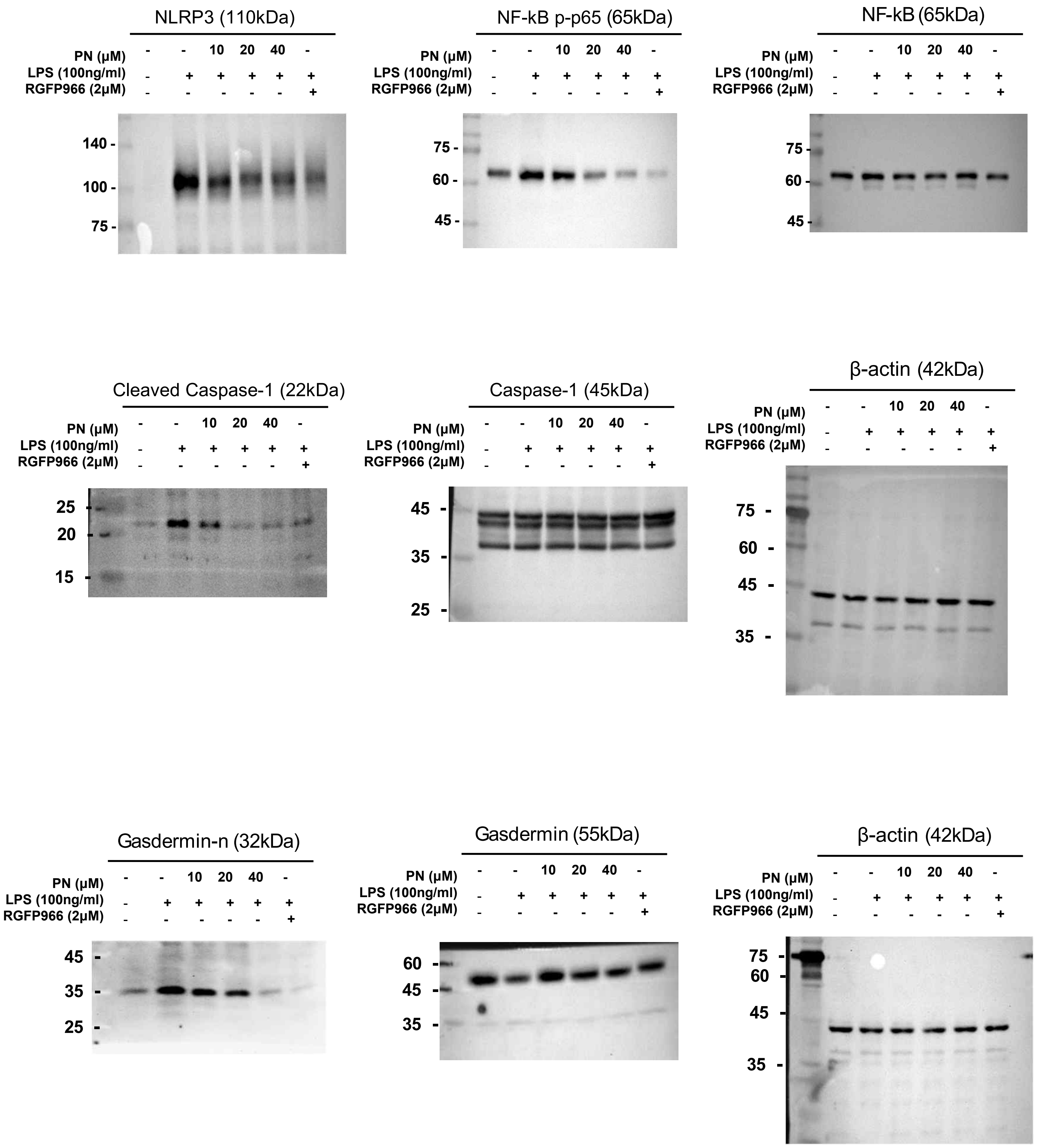

Fig.5C

MLE-12

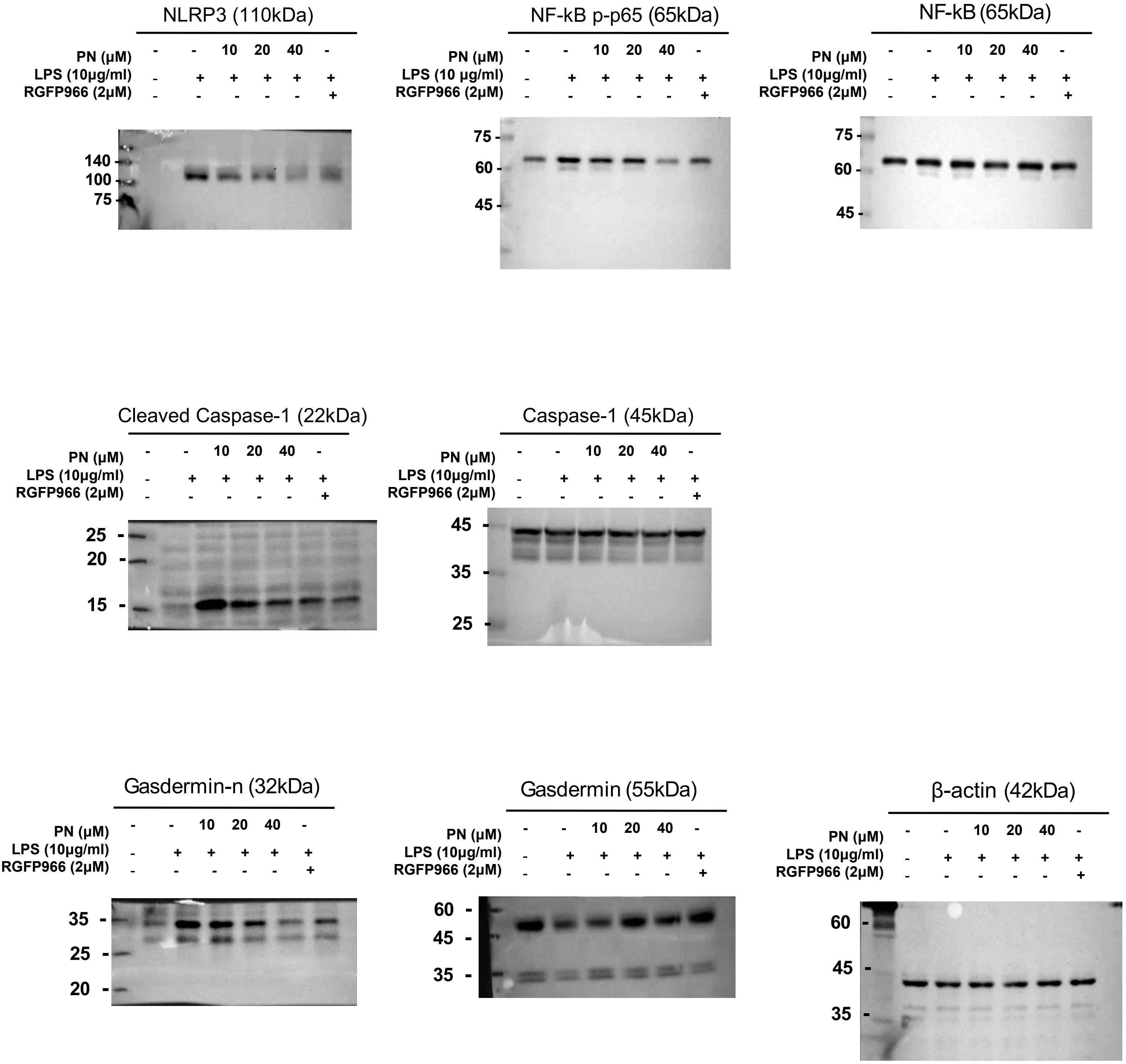
NLRP3 (110kDa)
PN (μM) - - 10 20 40 -
LPS (10μg/ml) - + + + + +
RGFP966 (2μM) - - - - - +
140
100
75
NF-kB p-p65 (65kDa)
PN (μM) - - 10 20 40 -
LPS (10 μg/ml) - + + + + +
RGFP966 (2μM) - - - - - +
75
60
45
NF-kB (65kDa)
PN (μM) - - 10 20 40 -
LPS (10μg/ml) - + + + + +
RGFP966 (2μM) - - - - - +
75
60
45
Cleaved Caspase-1 (22kDa)
PN (μM) - - 10 20 40 -
LPS (10μg/ml) - + + + + +
RGFP966 (2μM) - - - - - +
25
20
15
Caspase-1 (45kDa)
PN (μM) - - 10 20 40 -
LPS (10μg/ml) - + + + + +
RGFP966 (2μM) - - - - - +
45
35
25
Gasdermin-n (32kDa)
PN (μM) - - 10 20 40 -
LPS (10μg/ml) - + + + + +
RGFP966 (2μM) - - - - - +
35
25
20
Gasdermin (55kDa)
PN (μM) - - 10 20 40 -
LPS (10μg/ml) - + + + + +
RGFP966 (2μM) - - - - - +
60
45
35
β-actin (42kDa)
PN (μM) - - 10 20 40 -
LPS (10μg/ml) - + + + + +
RGFP966 (2μM) - - - - - +
60
45
35

Fig.7E

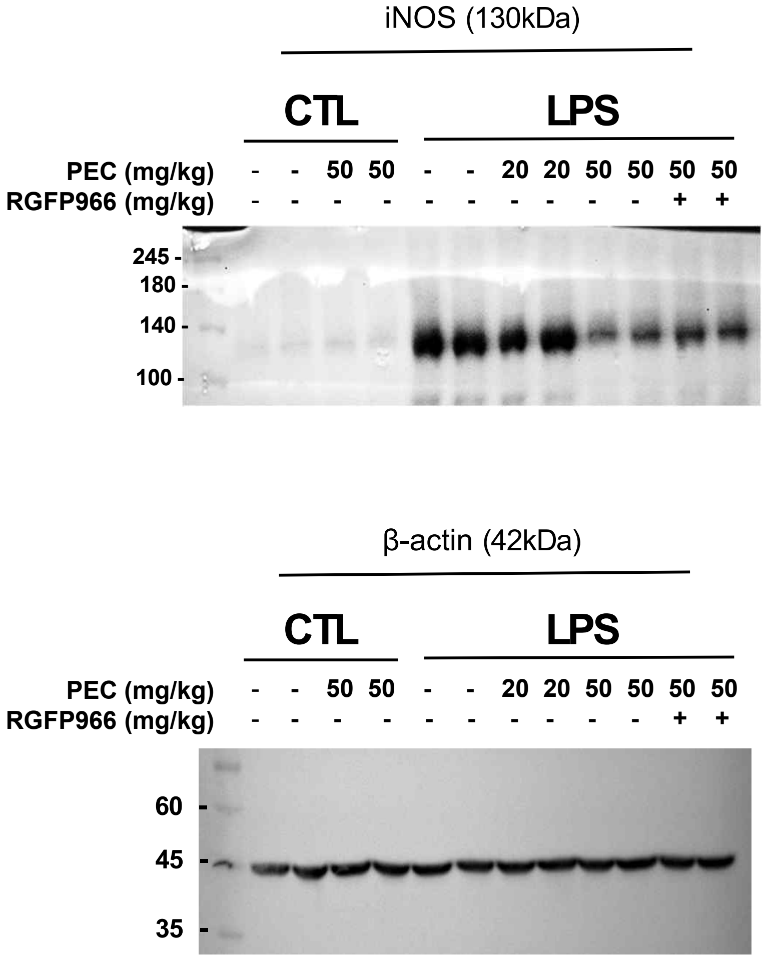

iNOS (130kDa)
CTL
LPS
PEC (mg/kg) - - 50 50 - - 20 20 50 50 50 50
RGFP966 (mg/kg) - - - - - - - - - - + +
245
180
140
100
β-actin (42kDa)
CTL
LPS
PEC (mg/kg) - - 50 50 - - 20 20 50 50 50 50
RGFP966 (mg/kg) - - - - - - - - - - + +
60
45
35

Fig.8A

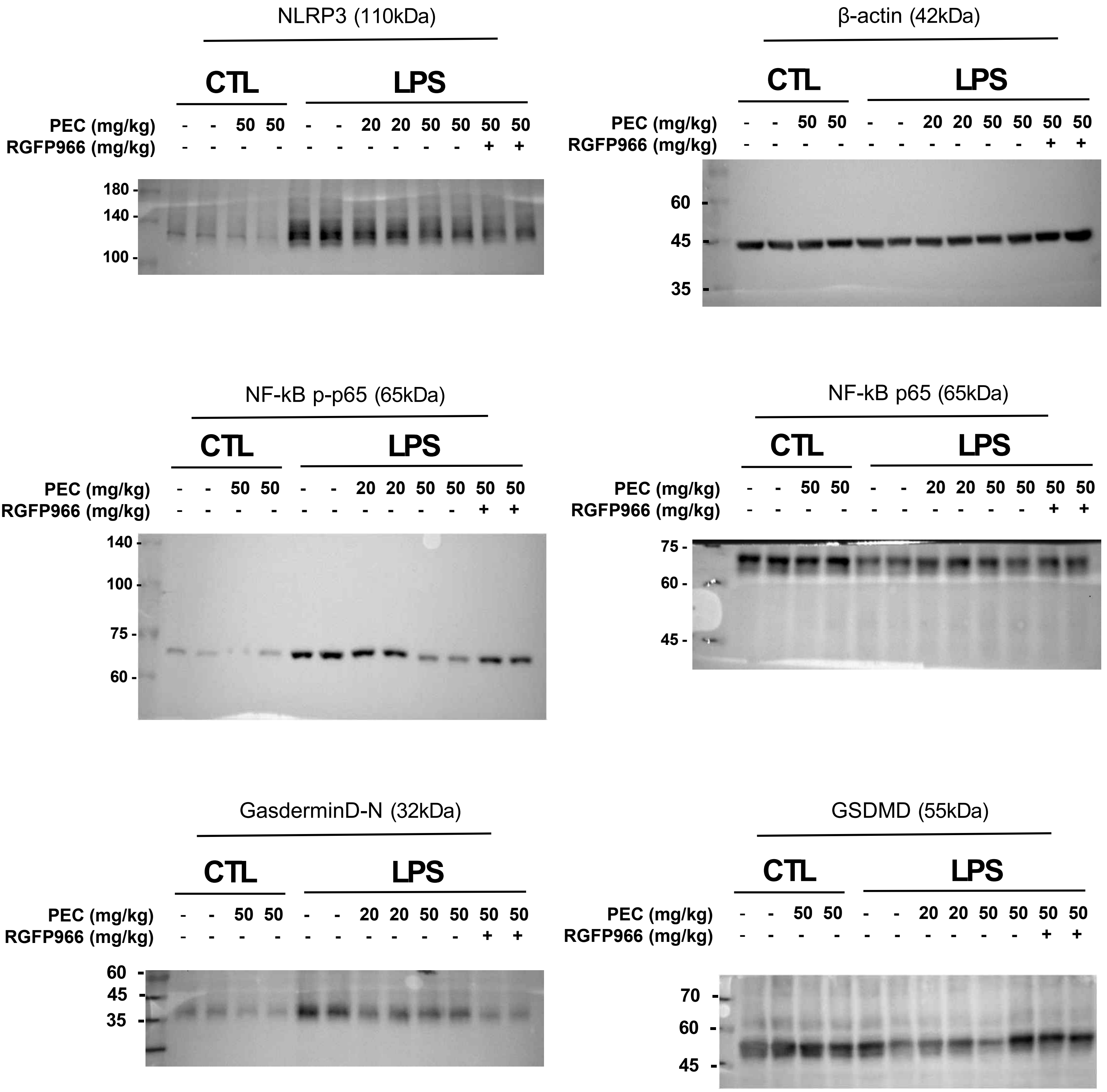
NLRP3 (110kDa)
CTL
LPS
PEC (mg/kg)
RGFP966 (mg/kg)
180
140
100
β-actin (42kDa)
60
45
35
NF-kB p-p65 (65kDa)
140
100
75
60
NF-kB p65 (65kDa)
75
60
45
GasderminD-N (32kDa)
60
45
35
GSDMD (55kDa)
70
60
45
