# Supplementary material for: Pectolinarigenin Attenuates LPS-Induced Lung Inflammation and Injury with Reduced HDAC3/NF-κB/NLRP3 Signaling
Source: Antioxidants (Basel). 2026 Jul 20;15(7):898. doi: 10.3390/antiox15070898 (PMC13404758; doi:10.3390/antiox15070898)
Supplement: Supplementary file 1 [file antioxidants-15-00898-s001.zip › Supplymentary Table_and_Figures.pdf]

Supplementary Table S1. Primary antibodies used for western blot analysis.

Antibody	Producer	Cat #	Host species	RRID	Dilution
NF-κB p65	Cell signaling	8242	Rabbit	AB_10860244	WB 1:1000
Phospho-NF-κB p65	Cell signaling	3033	Rabbit	AB_331284	WB 1:1000
IκBα	Santa Cruz	sc-1643	Mouse	AB_627772	WB 1:1000
Phospho-IκBα	Santa Cruz	sc-8404	Mouse	AB_627773	WB 1:500
iNOS	Abcam	ab178945	Rabbit	AB_2861417	WB 1:1000
COX2	Abclonal	A1253	Rabbit	AB_2759370	WB 1:1000
Lamin B1	Cell signaling	12586	Rabbit	AB_2650517	WB 1:1000
HDAC3	Abclonal	A2139	Rabbit	AB_2764158	WB 1:1000
NLRP3	Abclonal	A5652	Rabbit	AB_2766412	WB 1:1000
Caspase-1	Abclonal	A0964	Rabbit	AB_2757485	WB 1:1000
Cleaved Caspase-1	Cell signaling	89332	Rabbit	AB_2923067	WB 1:1000
NRF2	Abclonal	A21176	Rabbit	AB_3661892	WB 1:1000
Gasdermin D	Cell signaling	39754	Rabbit	AB_2916333	WB 1:1000
Cleaved Gasdermin D (A sp276) (N terminal)	Cell signaling	10137	Rabbit	AB_2923068	WB 1:1000
Acetyl-Histone H3-K27	Abclonal	A22264	Rabbit	AB_3698464	WB 1:1000
Histone H3	Abclonal	A22348	Rabbit	AB_3711666	WB 1:1000
β-Actin	Santa Cruz	sc-69879	Mouse	AB_1119529	WB 1:5000

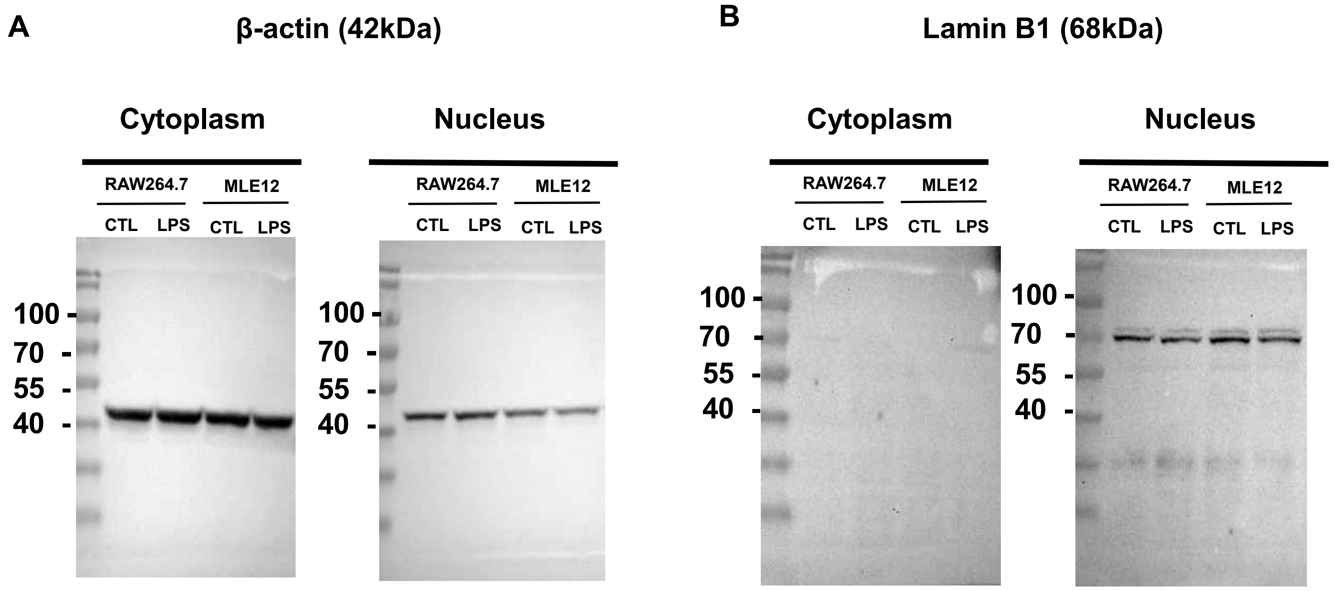

Supplementary Figure S1. Validation of nuclear and cytoplasmic fractionation purity.

The purity of the nuclear and cytoplasmic fractions was verified by Western blot analysis using markers for each compartment. (A) β-actin was used as a marker for the cytoplasmic fraction. Although a faint signal for β-actin was detected in the nuclear fraction, this is consistent with the known physiological role of nuclear actin. (B) Lamin B1 was used as a nuclear marker and was detected exclusively in the nuclear fraction, demonstrating the absence of nuclear contamination in the cytoplasmic fraction and the successful separation of the cellular components.

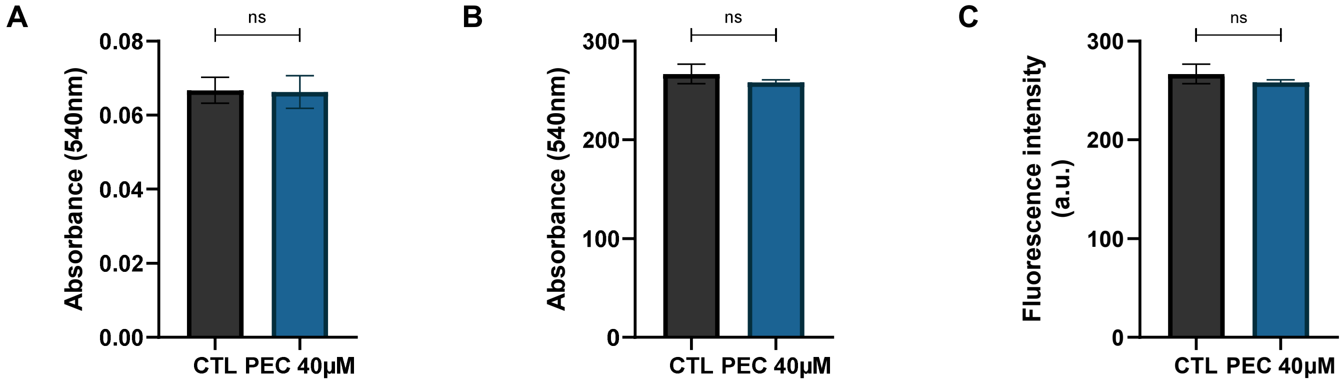

Supplementary Figure S2. Evaluation of potential assay interference by PEC using cell-free control experiments. (A) MTT, (B) Griess, and (C) DCF-DA assays were performed in a cell-free conditions to determine whether PEC directly interfered with the assay readouts. PEC was incubated with the assay reagents in the absence of cells under the same experimental conditions used for the biological assays. Data are presented as mean ± SD of three independent experiments performed in triplicate. Statistical significance was determined using Welch's t-test. ns, not significant. PEC, pectolinarigenin; CTL, control
